# Purification of human β- and γ-actin from budding yeast

**DOI:** 10.1242/jcs.260540

**Published:** 2023-05-09

**Authors:** Brian K. Haarer, Morgan L. Pimm, Ebbing P. de Jong, David C. Amberg, Jessica L. Henty-Ridilla

**Affiliations:** ^1^ Department of Biochemistry and Molecular Biology, State University of New York (SUNY) Upstate Medical University, Syracuse, NY 13210, USA; ^2^ Mass Spectrometry Core Facility; ^3^ Department of Neuroscience and Physiology, SUNY Upstate Medical University, Syracuse, NY 13210, USA

**Keywords:** Cytoplasmic actin, Non-muscle actin, β-actin, γ-actin

## Abstract

Biochemical studies of human actin and its binding partners rely heavily on abundant and easily purified α-actin from skeletal muscle. Therefore, muscle actin has been used to evaluate and determine the activities of most actin regulatory proteins but there is an underlying concern that these proteins perform differently from actin present in non-muscle cells. To provide easily accessible and relatively abundant sources of human β- or γ-actin (i.e. cytoplasmic actins), we developed *Saccharomyces cerevisiae* strains that express each as their sole source of actin. Both β- or γ-actin purified in this system polymerize and interact with various binding partners, including profilin, mDia1 (formin), fascin and thymosin-β4 (Tβ4). Notably, Tβ4 and profilin bind to β- or γ-actin with higher affinity than to α-actin, emphasizing the value of testing actin ligands with specific actin isoforms. These reagents will make specific isoforms of actin more accessible for future studies on actin regulation.

## Teaser

**Summary:** Generation and characterization of human β- and γ-actin from budding yeast as an accessible and low-cost way to study the regulation of specific actin isoforms.

## INTRODUCTION

**Fig. 1.**
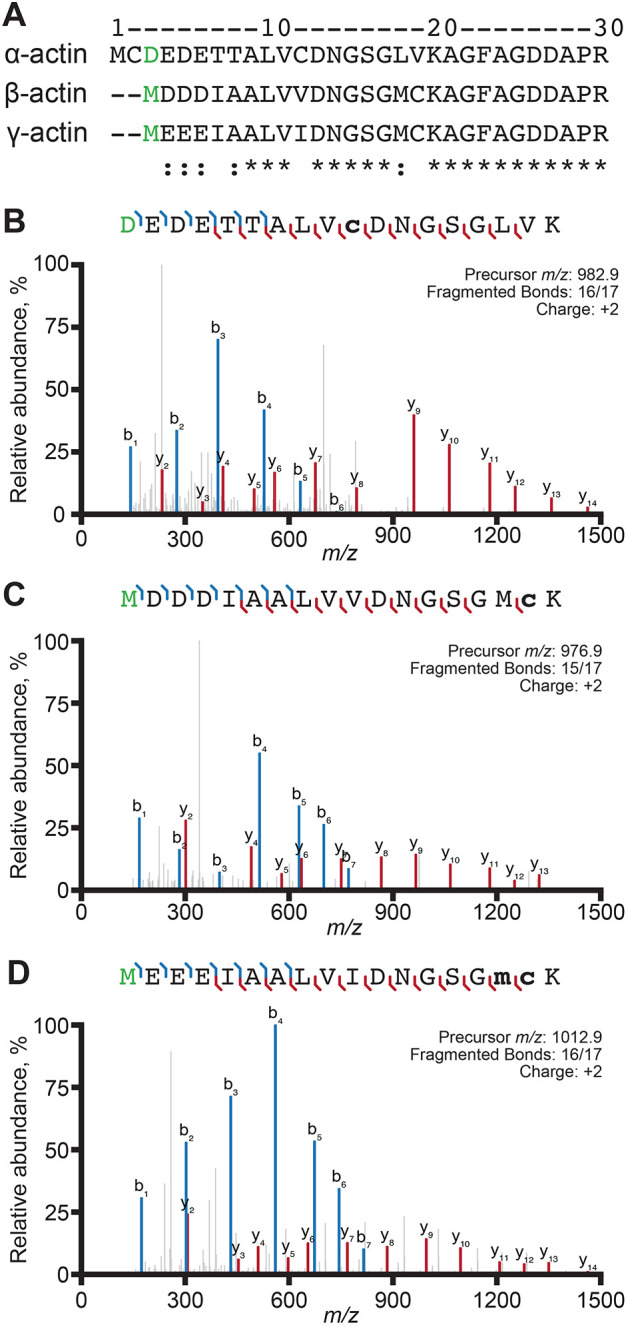
**β-actin and γ-actin are acetylated on the N-terminal methionine.** (A) Sequence alignment of human α1-actin, β-actin and γ1-actin. Spectra shown was performed (*n*=1) for acetylated (green) N-terminal peptides as indicated for (B) α-actin (RMA), (C) β-actin or (D) γ-actin. Bold lower-case letters: m, oxidized methionine; c, carbamidomethyl cysteine.

The actin cytoskeleton is an essential, highly conserved and abundant component of cells. Simple eukaryotes tend to express a single actin isoform, whereas humans display tissue and cell specific expression patterns. Although closely related, actin isoforms can subtly differ in biochemical properties related to polymer formation, nucleotide hydrolysis and exchange, and interactions with one or more essential regulatory proteins (Allen et al., 1996; Moradi et al., 2017; Namba et al., 1992; Perrin and Ervasti, 2010). Humans express six actin isoforms: α1, α2, α-cardiac and γ2 (encoded by *ACTA1, ACTA2*, *ACTC1* and *ACTG2*), which occur predominantly in skeletal, cardiac and smooth muscle cells, and β (*ACTB*) and γ1 (γ henceforth; *ACTG1*) found predominantly in non-muscle cells and considered cytoplasmic isoforms of actin. Human β- and γ-actin are structurally divergent (Arora et al., 2023), yet differ by only four amino acids located within their first ten amino acids (Fig. 1A). Various muscle tissues are the most common sources of actin for use in biochemical studies. Actin purified in this manner is present as a mixture of muscle isoforms and minor amounts of β- or γ-actin. Characterizing specific isoforms of actin has been limited by accessibility and is critically important for understanding mechanisms of disease (Parker et al., 2020). Thus, it has been challenging to discern the biochemical properties of either β- and γ-actin in biochemical assays.

Producing recombinant human actin outside of eukaryotic cells is difficult due to the complex network of chaperones needed to properly fold actin; however, several systems have been developed (Geissler et al., 1998; Grantham, 2020; Millán-Zambrano and Chávez, 2014; Schafer et al., 1998; Valpuesta et al., 2002). The pCold system permits the bacterial synthesis of recombinant tagged β-actin (Tamura et al., 2011). Actin isoforms expressed and purified from popular eukaryotic systems produce relatively large quantities of biochemically active actin (Bergeron et al., 2010; Bookwalter and Trybus, 2006; Ohki et al., 2009; Rutkevich et al., 2006; Yamashiro et al., 2014). Although these and related purification methods have been adopted for various studies of normal and mutant versions of actin, preparations are often contaminated with low amounts (5–15%) of host actin (Hundt et al., 2014; Müller et al., 2012, 2013; von der Ecken et al., 2016). Other systems use a combination of affinity tags and a direct fusion to the actin monomer-binding protein thymosin-β4 (Tβ4; also known as TMSB4) to prevent the spurious polymerization or aggregation of recombinant actin and to facilitate isoform specific purification (Mu et al., 2020; Hatano et al., 2018; Hatano et al., 2020; Kijima et al., 2016; Lu et al., 2015; Noguchi et al., 2007). With the addition of NAA80 or SETD3 enzymes, this approach permits the isolation of actin in specific post translationally modified states, including N-acetylation, N-arginylation and methylation of a conserved histidine residue (Arora et al., 2023; Hatano et al., 2018, 2020). Additional approaches even permit the close preservation of native secondary modifications (Ceron et al., 2022). Although each system requires post-purification processing, they provide a source of non-muscle, normal or mutant (including non-polymerizable), actin isoforms.

Here, we have taken a different approach to generate pure human β-or γ-actin, engineering the yeast *Saccharomyces cerevisiae* to produce either isoform as their only source of actin. This technique was first pioneered to purify chicken β-actin from yeast, relying on hydroxylapatite chromatography to separate host and recombinant actin (Karlsson, 1988). A follow-up study showed that yeast could survive, albeit not well, with this as their sole actin source, although β-actin was not purified from these yeast strains (Karlsson et al., 1991). We have taken codon-optimized genes for human β- and γ-actin and expressed them in yeast lacking the resident actin gene *ACT1*. The resulting strains grow considerably slower than wild-type yeast, yet provide significant, low-cost and easily obtainable yields of homogeneous β- or γ-actin. Although actin purified by this approach does not undergo standard mammalian N-terminal processing, each actin isoform polymerizes and interacts with several actin-binding proteins including profilin, Tβ4, the formin mDia1 (also known as DIAPH1) and fascin. Thus, these engineered strains provide convenient sources of pure β- or γ-actin for biochemical studies where conventional α-actin sources might be unsuitable.

## RESULTS

We expressed codon-optimized human cytoplasmic β- or γ-actin in *Saccharomyces cerevisiae* strains lacking the sole yeast actin gene (*ACT1*). The strain expressing human β-actin grew slowly (Fig. S1A) and displayed heterogeneous morphology (Fig. S1B–D), similar to what has been found for yeast expressing chicken β-actin (Karlsson et al., 1991). The strain expressing γ-actin grew even more slowly (Fig. S1A). However, both strains could be maintained without additional selection for the essential plasmid-borne actin genes. To confirm the identity and assess the posttranslational state of both isoforms, each recombinant actin was purified by affinity chromatography and subjected to mass spectrometry. This analysis showed 89% (β-actin), 95% (γ-actin) and 98% [α-actin; from rabbit muscle actin (RMA) as a control] coverage of the predicted peptide profiles. We took special note of the state of N-terminal peptides, which are the only peptides expected to differ between β- and γ-actin (Fig. 1A). We detected N-terminally acetylated β- and γ-actin (at Met1) (Fig. 1C,D), although not all intact peptides were N-acetylated. We also detected non-acetylated β- and γ-actin peptides truncated by one to five amino acids (Tables S1–S3) For comparison, all N-terminal peptides on α-actin (control) were truncated by two to six amino acids, present with and without acetylation on Asp3 (Fig. 1B; Tables S1–S3).

Two other important modifications of metazoan versions of actin include the methylation of His73, which might affect P_i_ release following ATP hydrolysis (Terman and Kashina, 2013), and Lys84, which might block myosin binding (Li et al., 2013). Each of these modifications were present in the α-actin (control) dataset but were absent in the β- and γ-actin spectra (Table S1). Finally, N-terminal arginylation of β-actin on Asp3 can influence cellular and biochemical functions (Chin et al., 2022; Karakozova et al., 2006). However, N-arginylation of β- or γ-actin on the third residue cannot be reliably distinguished from N-acetylation on the second residue (Tables S1 and S2) (Karakozova et al., 2006; Xu et al., 2009; Drazic et al., 2022). Not surprisingly, the modification profiles of β- and γ-actin reflect processing that normally occurs for yeast actin. Yeast processing includes the acetylation of N-terminal Met1 without further processing, and yeast lack additional methylating enzymes (Fig. 1; Tables S1 and S2; Kalhor et al., 1999).

**Fig. 2.**
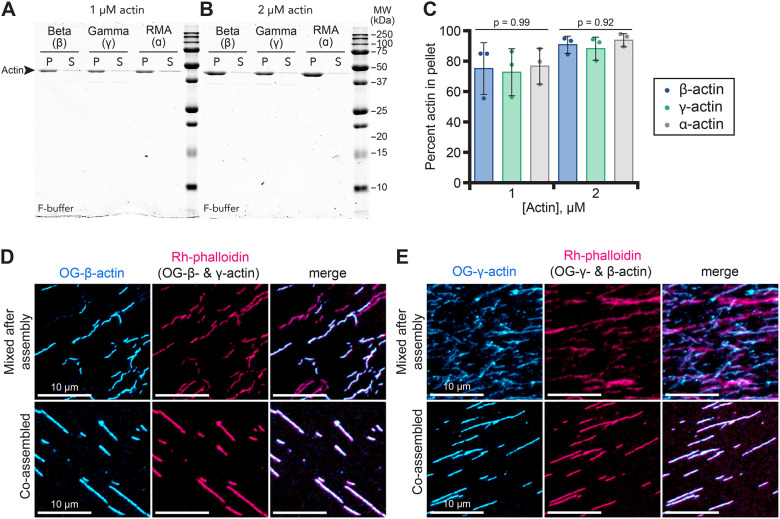
**Yeast-produced human β- and γ-actin form filaments and co-polymers.** (A,B) Representative gels of polymerized (P; pellet) or unpolymerized (S; supernatant) actin from pelleting assays performed with (A) 1 or (B) 2 µM actin. Additional uncropped gels are shown in Fig. S2. (C) Quantification by band densitometry of polymerized actin from pellet samples from experiments as in A and B (*n*=3 experiments). Statistics: one-way ANOVA with Tukey post test; values are not significantly different. (D) Top row, mixes of individually polymerized Oregon Green (OG)-labeled β-actin and unlabeled γ-actin stabilized with rhodamine (Rh)–phalloidin. Bottom row, co-polymerized OG-β-actin and unlabeled γ-actin, stained with Rh–phalloidin. (E) Similar reactions as in D with OG-labeled γ-actin and unlabeled β-actin stained with Rh–phalloidin. Images in D and E representative of *n*>3 fields of view. Scale bars: 10 µm.

**Fig. 3.**
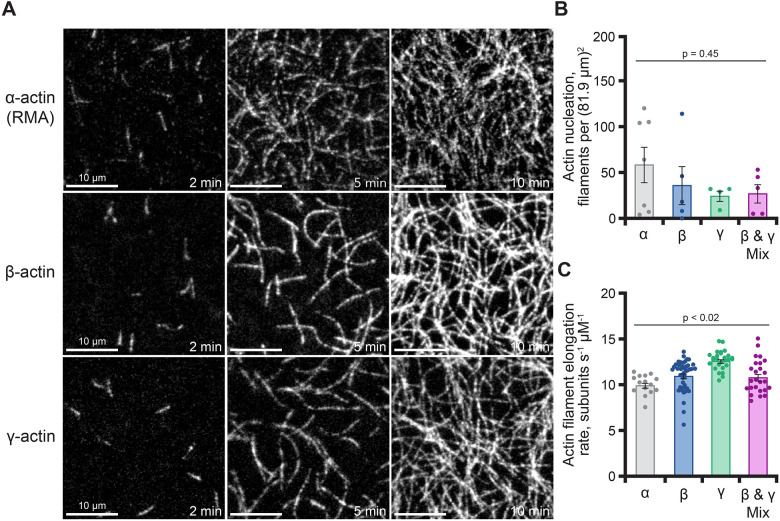
**Actin isoforms display similar phases of actin assembly.** (A) Images from time-lapse TIRF microscopy reactions containing 1 µM total actin monomers as indicated (10% fluorescently labeled RMA). Scale bars: 10 µm. See Movie 1. (B) Actin nucleation (*n*=3–6; mean±s.e.m.) 100 s after the initiation of filament polymerization from reactions as in A. (C) Mean±s.e.m. actin elongation rates for individual filaments (*n*=15–45 per condition from at least three different reactions; dots) present in reactions as in A. *P*-values presented are from one-way ANOVA tests with Tukey post test across conditions (all data presented under the line). Only comparisons to γ-actin elongation rates were significantly different [each comparison was *P*<0.01 for γ-actin compared to α-actin, β-actin, and the reaction containing a mixture of β- and γ-actin (β and γ-mix)]. Comparisons between α- and β-actin were not statistically different in this dataset.

The ability of both β- and γ-actin to support yeast growth suggests protein functionality (Fig. S2). To explore this idea further, we used classic biochemical assays to characterize each cytoplasmic actin. We began by assessing the ability of β- and γ-actin to polymerize into filaments with conventional pelleting assays (Fig. 2A,B). Polymerization of each actin was indistinguishable, with filaments pelleting to similar degrees at multiple concentrations (Fig. 2A–C). Each actin remained in the supernatant under controls performed in G-buffer (Fig. S2). To assess whether β- and γ-actin could co-polymerize, we performed epifluorescence microscopy to visualize actin filaments polymerized before or after mixing unlabeled and Oregon Green (OG)-labeled β- or γ-actin bound to rhodamine (Rh)–phalloidin (Fig. 2D,E). Rh–phalloidin stabilization of actins polymerized before mixing resulted in a mixed population of filaments including Rh–phalloidin-decorated unlabeled actin (only visible in the Rh–phalloidin channel) and OG-labeled filaments co-labeled with Rh–phalloidin (visible in both channels) (Fig. 2D,E, top rows). In contrast, mixing of actins prior to polymerization, followed by Rh–phalloidin stabilization, produced uniformly labeled actin filaments in both wavelengths, consistent with co-polymerization (Fig. 2D,E, bottom rows). Notably, filament co-labeling occurred regardless of which actin was OG labeled, consistent with co-polymerization of β- and γ-actin. To further assess the actin assembly properties of β- and γ-actin, we used total internal reflection fluorescence (TIRF) microscopy to directly observe single actin filament polymerization (Fig. 3A; Movie 1). Unlabeled 1 µM β-actin, γ-actin or control α-actin (RMA) was visualized with 10% fluorescently labeled RMA (Fig. 3A) (Chin et al., 2022; Hatano et al., 2018). Under these conditions, α-, β- or γ-actin showed similar means of nucleation ranging from 25.5±5.5 to 59.7±19.3 filaments per field of view (mean±s.e.m.; *P*≥0.45) (Fig. 3B). Control α-actin filaments (RMA) elongated at a rate of 10.0±0.3 subunits s^−1^ µM^−1^ (mean±s.e.m.), consistent with other studies (Fig. 3C) (Liu et al., 2022; Pimm et al., 2022). β-actin and γ-actin filaments polymerized at mean rates of 11.1±0.3 subunits s^−1^ µM^−1^ and 12.7±0.2 subunits s^−1^ µM^−1^, respectively (Fig. 3C). The mean elongation rate for γ-actin filaments was significantly faster than α-actin, β-actin or a 1:1 mixture of cytoplasmic isoforms (10.9±0.4 subunits s^−1^ µM^−1^; *P*=0.02) (Fig. 3C).

**Fig. 4.**
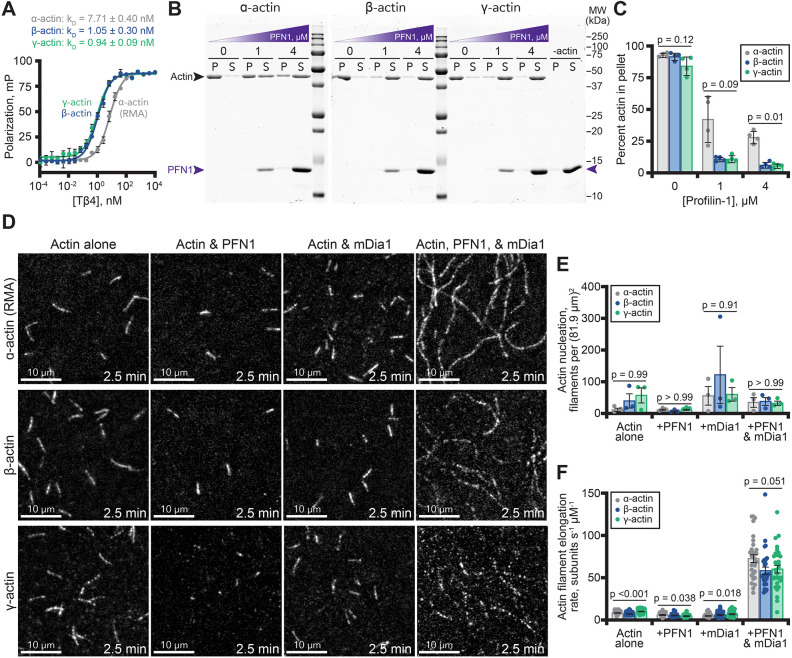
**Polymerization of β- or γ-actin with actin assembly factors.** (A) Fluorescence polarization of GFP–Tβ4 mixed with 10 nM unlabeled α-actin (RMA; gray), β-actin (blue) or γ-actin (green). Curves shown are average fits from *n*=3 separate experiments. Dots are mean±s.e.m. (B) Representative gels displaying the fraction of polymerized (P; pellet) or unpolymerized (S; supernatant) actin present in pelleting assays performed with 2 µM actin and different concentrations of profilin (PFN1; purple arrow). Additional uncropped gels shown in Fig. S3. (C) Quantification by densitometry of polymerized actin from pellet samples from B. Mean±s.e.m. (*n*=4). (D) TIRF images from reactions containing 1 µM actin (10% Alexa Fluor 488-conjugated RMA) and combinations of 2 µM PFN1 and 10 nM mDia1(FH1-C), as indicated, at 2.5 min after addition. Scale bars: 10 µm. See Movie 2. (E) Actin nucleation (mean±s.e.m.; *n*=3 fields of view; dots) 100 s after the initiation of filament polymerization from reactions as in D. (F) Mean±s.e.m. actin elongation rates for individual filaments (*n*=30–45 per condition from at least three different reactions; dots) present in reactions as in D. *P*-values presented are from one-way ANOVA with Tukey post test comparing all conditions; see Fig. S4 for a rescaled plot and additional comparisons of the data presented in F.

Cellular functions of actin are further modulated through interactions with many regulatory proteins. Therefore, we assessed the activities of several classic regulators of actin polymerization dynamics in the presence of either actin isoform. In mammalian cells, Tβ4 sequesters actin monomers to regulate available subunits for filament polymerization (Skruber et al., 2018, 2020). We assessed each human actin made in yeast for Tβ4 binding using fluorescence polarization (Fig. 4A). GFP–Tβ4 bound to β-actin [*K*_D_=1.05±0.30 nM (mean±s.e.m.)] and γ-actin (*K*_D_ =0.94±0.09 nM) with similar affinities, which were each significantly stronger than skeletal muscle actin (*K*_D_=7.71 nM±0.40; *P*<0.002) (Fig. 4A) (Pimm et al., 2022). This reinforces the notion that actin-binding proteins may have different functions with specific isoforms of actin.

To explore this idea further, we assessed the activities of profilin with each isoform of actin. Profilin directly inhibits α-actin assembly (Ferron et al., 2007; Pimm et al., 2020; Skruber et al., 2018). We began using pelleting assays to assess whether profilin (referring to profilin-1, PFN1) preferentially blocked the assembly of β- or γ-actin filaments (Fig. 4B). Profilin bound each actin isoform and strongly reduced signals in the pellet fractions of each cytoplasmic actin. We noted that profilin blocked the assembly of β- and γ-actin more effectively than muscle actin (*P*≤0.01) (Fig. 4C) (Antoku et al., 2019; Kinosian et al., 2000). However, no significant differences were observed for profilin-mediated nucleation in TIRF assays (*P*>0.99) (Fig. 4D,E).

When present with the actin polymerization-stimulating formin (mDia1), profilin enhances actin filament elongation (Chesarone et al., 2010; Li and Higgs, 2003; Valencia and Quinlan, 2021). Thus, to assess whether β- or γ-actin promotes formin-based filament assembly, we used TIRF microscopy to monitor actin filament polymerization in the presence of the constitutively active formin mDia1(FH1-C). Each actin isoform stimulated nucleation to similar levels (*P*>0.91) (Fig. 4D,E) and stimulated actin filament elongation when combined with formin and profilin (Fig. 4D,F; Fig. S4A–C), with γ-actin filaments elongating significantly faster than the other isoforms (see Fig. S4 for all statistical comparisons and rescaled plots). Compared to α-actin filaments, which grew at 73.6±4.5 subunits s^−1^ µM^−1^ (mean±s.e.m.), the mean elongation rate for β-actin and γ-actin with profilin and formin was not significantly slower at 59.3±4.2 or 61.2±4.5 subunits s^−1^ µM^−1^, respectively (*P*≤0.051) (Fig. 4F; Fig. S4A–C, Movie 2). In summary, α-actin from muscle and both cytoplasmic actins display similar properties in the presence of profilin and the formin mDia1, albeit to different levels.

**Fig. 5.**
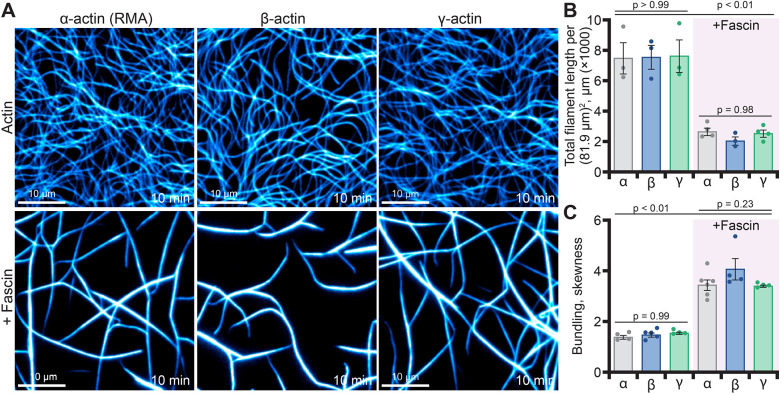
**Fascin-mediated actin filament bundling of actin isoforms.** (A) Representative TIRF images from reactions containing 1 µM unlabeled actin visualized with Alexa-488 phalloidin alone or in the presence of 1 µM fascin. Scale bars: 10 µm. (B) Total actin polymer (ridge detection analysis) from reactions as in A; mean±s.e.m., *n*=3–4 fields of view (FOV; dots). (C) Filament bundling (skewness) from reactions as in A; mean±s.e.m., *n*=4–6 FOV (dots). *P*-values presented are from one-way ANOVA with Tukey post test; the only statistical differences found were between untreated controls and fascin-treatments but not between actin isoforms.

Finally, the higher-order organization of cellular actin arrays is commonly achieved through the association of proteins that cross-link or bundle filaments. We produced actin bundles with fascin, stained filaments with fluorescent phalloidin and used TIRF microscopy to compare the higher-order structures made by each isoform (Fig. 5A). As actin filaments bundle, the overall area covered by pixel signal decreases (Fig. 5A). Meanwhile, the distribution of pixel intensities shifts to brighter pixels (Higaki et al., 2010; Khurana et al., 2010). Thus, we quantitatively measured the extent of actin filament bundling using length and intensity-based metrics to assess total bundling (Fig. 5B,C). As actin filaments coalesced into bundles, the number of objects detected in ridge analysis decreased to similar levels, regardless of actin isoform (*P*=0.98) (Fig. 5B). In contrast, the fluorescence intensity of β-, γ- and α-actin filaments crosslinked by fascin were similar to each other but each was significantly brighter than fascin-absent controls (*P*<0.01) (Fig. 5C). In conclusion, this demonstrates actin filaments of each isoform can be bundled by fascin.

## DISCUSSION

We developed budding yeast strains for the expression and purification of human cytoplasmic β- and γ-actin, and have demonstrated that these actin isoforms polymerize and interact with several canonical actin-binding proteins. There are several benefits to purifying recombinant actin from this system including: yield (0.5–1 mg/l starting culture), no special growth or tissue culture requirements, use of conventional purification reagents and protocols, no additional post-purification processing (e.g. cleavage of fusion tags), and no contaminating ‘host’ actin. Even with these advantages, we note that for both yeast and human actin purifications, a significant amount of protein (>50%) is lost following post-elution dialysis. This likely reflects the denaturing activity of the formamide used to elute actin from the DNaseI column and might be improved by supplementing our approach with an alternative 6×His–gelsolin fragment-based purification (Ceron et al., 2022; Ohki et al., 2009).

Comparisons of actin isoforms or mixes that reflect other species and non-muscle cells are crucial to our understanding of how actin and its regulators truly function. For example, the formin FHOD1 displays differential interactions with actin from different sources, specifically enhancing the nucleation phase of actin assembly with cytoplasmic actin but preventing the assembly of filaments generated from RMA (Antoku et al., 2019; Patel et al., 2018). Profilin and cofilin proteins each bind cytoplasmic actin with higher affinity than to muscle actin (Antoku et al., 2019; De La Cruz, 2005; Kinosian et al., 2000). Furthermore, specific myosin motors differentially prefer muscle or non-muscle sources, some even preferring specific β- over γ-actin isoforms for movement (Müller et al., 2013). Our results identify Tβ4 as an additional actin regulatory protein influenced by actin source, binding β- or γ-actin with higher affinity than to actin purified from muscle.

Over 100 post-translational modifications (PTMs) of cytoplasmic and muscle actins have been described, with common reports of acetylation, arginylation, methylation and phosphorylation (Mu et al., 2020; Terman and Kashina, 2013; Varland et al., 2019). Post-translationally modified actin might influence the rate of polymerization, filament–filament interactions, actin-binding protein interactions, cellular localization and locomotion (Arnesen et al., 2018; Varland et al., 2019; Yamashiro et al., 2014). Perhaps most relevant for the discussion of cytoplasmic actin isoforms is N-terminal acetylation, as β- and γ-actin differ by four residues in this region (Fig. 1A). We found that yeast-produced β- and γ-actin were each acetylated on the N-terminal methionine (Tables S1–S3) (Cook et al., 1991). One notable missing modification from actin purified in this system is methylation of His73, which is important for the nucleotide-exchange of actin (Wilkinson et al., 2019). Introducing NAA80 or SETD3 to this system might ameliorate these challenges and expand the versatility of our budding yeast-based purification system (Arora et al., 2023; Hatano et al., 2020).

Most mammalian actin is processed to remove the N-terminal methionine and then acetylated or less commonly (and only for β-actin) arginylated at Asp3 (Varland et al., 2019). Studies in yeast and other systems further suggest that N-terminal arginylation occurs on clipped or deacetylated proteins, which might target them for proteasomal degradation (Drazic et al., 2022; Kumar et al., 2016; Nguyen et al., 2019). N-terminal differences in actin isoforms might be important for some applications and less consequential for others (Cook et al., 1992; Hatano et al., 2018). Notably, several studies have successfully purified and utilized versions of human actin lacking N-terminal modifications other than truncations (Mu et al., 2020; Lu et al., 2015). For those utilizing muscle actin to study interactions with non-muscle actin-binding proteins, differences between actin isotypes (primary amino acid composition) might prove more important than the exact nature of secondary modifications. Our system provides easily obtainable and relatively inexpensive sources of bulk cytoplasmic actin that will work well for many applications and that can be useful partners to systems that allow tighter control of specific PTMs or the synthesis of mutated versions of actin (Ceron et al., 2022; Hatano et al., 2020).

## MATERIALS AND METHODS

### Reagents and supplies

Unless otherwise specified, chemicals and supplies were purchased from Thermo Fisher Scientific (Pittsburgh, PA). DNaseI for affinity columns was purchased from Worthington Biochemical (Lakewood, NJ). Cloning reagents were obtained as follows: restriction enzymes and DNA ligase (New England Biolabs; Ipswich, MA); Primestar HS DNA polymerase (Takara Bio USA; San Jose, CA); and oligonucleotides (Eurofins Genomics; Louisville, KY).

### Plasmid and strain construction

Human α1-actin (*ACTA1*; NCBI Gene ID: 58) and β-actin (*ACTB*; NCBI Gene ID: 60) genes flanked by BamHI and HindIII sites were codon-optimized for expression in budding yeast (GenScript; Piscataway, NJ). Codon-optimized γ1-actin (*ACTG1*; NCBI Gene ID: 71) was generated from the ACTB sequence with the following mutagenic primer (5′-CTCGGATCCATGGAAGAAGAAATTGCTGCATTGGTTATTGATAATGGTTCTGGCATGTG-3′). The TDH3 promoter was PCR amplified as an EcoRI-BamHI fragment from yeast genomic DNA with 5′-GTGAGAATTCTCAGTTCGAGTTTATCATTA-3′ and 5′-CTCGGATCCTTTGTTTGTTTATGTGTGTTT-3′. TDH3 and actin genes were inserted into YEp351 (Hill et al., 1986). The yeast strain expressing only β-actin (BHY845) is a haploid segregant of an *ACT1/act1*Δ heterozygous diploid that carried the β-actin expression plasmid. The yeast strain that expresses only γ1-actin (BHY848) was generated by introducing the γ1-actin expression plasmid into a haploid *act1*Δ strain carrying the yeast *ACT1* gene on a *URA3*-based plasmid (Haarer et al., 2007). We used 5-fluoroorotic acid (FOA) to select for yeast that lost the *ACT1* (*URA3*) plasmid but remained viable. We were unable to recover yeast expressing human α1-actin, consistent with previous reports (McKane et al., 2005, 2006). We re-isolated and sequenced plasmids from β- and γ-actin strains at the time of harvest to confirm sequence fidelity. Each actin gene sequence was identical to the starting yeast-optimized *ACTB* and *ACTG1* genes.

### Protein purification

Human β- and γ-actin were prepared as described in Aggeli et al. (2014), with the following modifications: yeast were grown in 1 l of 1% yeast extract, 2% bactopeptone and 2–4% glucose (YPD), harvested by centrifugation at 8000 ***g*** for 7 min, washed in 25 ml 10 mM Tris-HCl pH 7.5, 0.2 mM CaCl_2_. Pellets were collected by centrifugation at 4000 ***g*** for 10 min and stored frozen at −80°C. Pellets were suspended in 20 ml G-buffer (10 mM Tris-HCl pH 7.5, 0.2 mM CaCl_2_, 0.5 mM ATP and 0.2 mM DTT) supplemented with protease inhibitors [0.1 mM PMSF and 1:500 Cocktail IV (Calbiochem; San Diego, CA)] and lysed by two passes through a French press at 1000 psi (6.9 MPa). Lysates were bound to 3 ml DNaseI–Sepharose, then beads were washed in five column volumes of each of the following: (1) 10% formamide in G-buffer, (2) 0.2 M NH_4_Cl in G-buffer, and (3) G-buffer alone. Actin was eluted with 50% formamide in G-buffer, and then immediately diluted with 1–2 ml G-buffer and dialyzed overnight against 2 l G-buffer. To remove contaminating proteins, actin underwent at least two polymerization–depolymerization cycles alternating between 0.6 M KCl and pipetting with dialysis against G-buffer.

Rabbit muscle actin (RMA; α-actin), mDia1(FH1-C) (amino acids 571–1255), GFP–Tβ4, profilin-1 and fascin used in TIRF or anisotropy assays were purified as described previously (Jansen et al., 2011; Liu et al., 2022; Pimm et al., 2022). Actin was labeled with Oregon Green iodoacetamide or Alexa Fluor NHS-Ester (Aggeli et al., 2014; Hertzog and Carlier, 2005; Kuhn and Pollard, 2005). Proteins were aliquoted, flash-frozen in liquid nitrogen and stored at −80°C. Proteins used in TIRF assays are shown in Fig. S5.

### Mass spectrometry analysis

Sample preparation, operation and mass spectrometry (MS) analyses were performed by the Upstate Medical University Proteomics and Mass Spectrometry Core Facility. Samples (15–30 µg) were treated with 5 mM tris(2-carboxyethyl)phosphine (TCEP) and then 15 mM iodoacetamide for 30 min in the dark. Trypsin digestion was performed at 37°C with 0.66 µg Trypsin Platinum (Promega, Madison, WI) overnight. Samples were acidified and then desalted using 2-core MCX stage tips (Rappsilber et al., 2003). Peptides were eluted with 75 µl of 65% acetonitrile (ACN) with 5% ammonium hydroxide, and dried. Samples were dissolved in 65 µl 2% ACN and 0.5% formic acid in water, then 0.5 µg was injected onto a pulled tip nano-LC column held at 50°C, with 100 µm inner diameter packed to 32 cm with 1.9 µm, 100 Å, core shell Magic2 C18AQ particles (Premier LCMS, Penn Valley, CA). Peptides were separated with a 3–28% ACN gradient over 60 min, followed by an increase to 85% ACN over 5 min. The inline Orbitrap Lumos was operated at 2.3 kV in data-dependent mode with a cycle time of 2.5 s. MS1 scans were collected at 120,000 resolution with a maximum injection time of 50 ms. Dynamic exclusion was applied for 15 s. Stepped HCD fragmentation of 34, 38 and 42% collision energy was used followed by two MS2 microscans in the Orbitrap at 15,000 resolution with dynamic maximum injection time.

The core facility used SequestHT in Proteome Discoverer (version 2.4; Thermo Fisher Scientific) to search three MS databases: *S. cerevisiae* (Uniprot, 6816 entries, retrieved 2017), Rabbit *O. cuniculus* (Uniprot, 41462 entries, retrieved 2023), a list of common contaminants (Thermo Fisher Scientific, 298 entries), and a custom list of human β-actin, γ-actin and rabbit α-actin. Additional entries for each actin were made with truncations of the first one, two and three N-terminal residues. Enzyme specificity was set to semi-tryptic with up to two missed cleavages. Precursor and product ion mass tolerances were set to 10 ppm and 0.02 Da. Cysteine carbamidomethylation was set as a fixed modification and the following modifications were set as variable: methionine oxidation, protein N-terminal acetylation, peptide N-terminal arginylation, lysine acetylation or methylation of lysine, histidine and arginine. The output was filtered using the Percolator algorithm. We visualized datasets with software at http://www.interactivepeptidespectralannotator.com/PeptideAnnotator.html (Brademan et al., 2019). Full data sets have been deposited at the PRIDE Database (ProteomeXchange identity: PXD040174).

### F-actin sedimentation assays

Polymerization of 1 or 2 µM actin was induced with the addition of concentrated (20×) F-buffer (final 1× concentration: 10 mM Tris (pH 7.5), 25 mM KCl, 4 mM MgCl_2_, 1 mM EGTA, 0.5 mM ATP). Samples were incubated for 30 min at room temperature, then subjected to centrifugation at 200,000 ***g*** for 30 min at 20°C. Supernatants were removed and pellets and supernatant samples were brought to equal volumes in protein loading buffer. Equal volumes were loaded on SDS-polyacrylamide gels and stained with Coomassie Brilliant Blue. Indicated concentrations of profilin-1 were mixed with 2 µM actin monomers in G-buffer and then incubated for 30 min at room temperature. Actin polymerization was then induced with F-buffer for an additional 30 min prior to centrifugation as above.

### Fluorescence-based actin polymerization assays

Actin isoform co-polymerization reactions were assessed by epifluorescence microscopy (Zeiss Imager.Z1, Oberkochen, Germany). 2 µM unlabeled or OG-labeled β- or γ-actin were polymerized in F-buffer, either individually or as a 1:1 mixture, then bound to 1.1 µM rhodamine–phalloidin. Samples were viewed individually or as mixtures bound to phalloidin prior to mixing. Samples were flowed into slide chambers prepared by laying a cover glass onto a slide with two intervening strips of double-sided tape, then visualized with DsRed and FITC filter sets. Views were obtained near fluid–air boundaries.

### TIRF microscopy assays

We prepared and visualized TIRF imaging chambers on a DMi8 TIRF microscope (Leica Microsystems; Wetzlar, Germany) as in Henty-Ridilla (2022), with the following modifications: reactions were executed in a different TIRF buffer [20 mM imidazole pH 7.4, 50 mM KCl, 1 mM MgCl_2_, 1 mM EGTA, 0.2 mM ATP, 10 mM DTT, 40 mM glucose and 0.25% methylcellulose (4000 cP)], with minimal (5–7%) labeled RMA. Some TIRF experiments with β- or γ-actin were supplemented with 10% RMA to enable visualization of filaments, as noted in figure legends. Experiments performed with fascin used unlabeled actin isoforms and were visualized with 130 nM Alexa Fluor 488-conjugated phalloidin. Filament elongation rates (subunits s^−1^ µM^−1^) were determined by measuring filament lengths from at least five frames, with a conversion factor of 370 subunits/µm (Kuhn and Pollard, 2005). Total filament or bundle length was calculated using the FIJI Ridge Detection plugin with settings that minimized background signals but permitted the detection of faint filaments without image saturation and applied identically to all images (Steger, 1998; Wagner et al., 2017). The skewness parameter was measured from FIJI measurements (Higaki et al., 2010; Khurana et al., 2010; Schindelin et al., 2012).

### Fluorescence polarization assays

Fluorescence polarization determination of GFP–Tβ4 binding to actin (Liu et al., 2022) was carried out in 1× PBS (pH 8.0) supplemented with 150 mM NaCl. 10 nM β- or γ-actin were mixed with various concentrations (0.1 pM to 10 µM) of GFP–Tβ4 and incubated at room temperature for 15 min. Fluorescence polarization was measured in a plate reader equipped with a monochromator, with excitation at 440 nm and emission intensity detection at 510 nm (bandwidth set to 20 nm) (Tecan; Männedorf, Switzerland). Three technical replicates were carried out on the same plate.

### Data analysis, statistics and availability

GraphPad Prism (version 9.5.0; GraphPad Software, San Diego, CA) was used to plot all data and perform statistical tests. All experiments were repeated at least three times. Individual data points are presented as dots in each figure and histograms represent mean±s.e.m. (unless noted otherwise). All one-way ANOVA tests performed compared all means with Tukey post-hoc analysis and passed tests for normality. *P*-values presented are those from ANOVA tests across tested conditions (under the line), except for Fig. S4 where all comparisons are listed. The threshold (*P*≤0.05) was used to determine significance throughout this work. Specific comparisons are described in each figure legend. Figures were made in Adobe Illustrator 2023 (version 27.1.1; Adobe, San Jose, CA).

## Supplementary Material

10.1242/joces.260540_sup1 Supplementary information

## References

[JCS260540C1] Aggeli, D., Kish-Trier, E., Lin, M. C., Haarer, B., Cingolani, G., Cooper, J. A., Wilkens, S. and Amberg, D. C. (2014). Coordination of the filament stabilizing versus destabilizing activities of cofilin through its secondary binding site on actin. *Cytoskeleton* 71, 361-379. 10.1002/cm.21178 24943913 PMC4241054

[JCS260540C2] Allen, P. G., Shuster, C. B., Kas, J., Chaponnier, C., Janmey, P. A. and Herman, I. M. (1996). Phalloidin binding and rheological differences among actin isoforms. *Biochemistry* 35, 14062-14069. 10.1021/bi961326g 8916891

[JCS260540C3] Antoku, S., Wu, W., Joseph, L. C., Morrow, J. P., Worman, H. J. and Gundersen, G. G. (2019). ERK1/2 Phosphorylation of FHOD Connects signaling and nuclear positioning alternations in cardiac laminopathy. *Dev. Cell* 51, 602-616.e612. 31794718 10.1016/j.devcel.2019.10.023 PMC7561008

[JCS260540C4] Arnesen, T., Marmorstein, R. and Dominguez, R. (2018). Actin's N-terminal acetyltransferase uncovered. *Cytoskeleton* 75, 318-322. 10.1002/cm.21455 30084538 PMC6226318

[JCS260540C5] Arora, A. S., Huang, H.-L., Singh, R., Narui, Y., Suchenko, A., Hatano, T., Heissler, S. M., Balasubramanian, M. K. and Chinthalapudi, K. (2023). Structural insights into actin isoforms. *eLife* 12, e82015. 10.7554/eLife.82015 36790143 PMC10072879

[JCS260540C6] Bergeron, S. E., Zhu, M., Thiem, S. M., Friderici, K. H. and Rubenstein, P. A. (2010). Ion-dependent polymerization differences between mammalian beta- and gamma-nonmuscle actin isoforms. *J. Biol. Chem.* 285, 16087-16095. 10.1074/jbc.M110.110130 20308063 PMC2871477

[JCS260540C7] Bookwalter, C. S. and Trybus, K. M. (2006). Functional consequences of a mutation in an expressed human alpha-cardiac actin at a site implicated in familial hypertrophic cardiomyopathy. *J. Biol. Chem.* 281, 16777-16784. 10.1074/jbc.M512935200 16611632

[JCS260540C8] Brademan, D. R., Riley, N. M., Kwiecien, N. W. and Coon, J. J. (2019). Interactive peptide spectral annotator: a versatile web-based tool for proteomic applications. *Mol. Cell Proteomics* 18, S193-S201. 10.1074/mcp.TIR118.001209 31088857 PMC6692776

[JCS260540C9] Ceron, R. H., Carman, P. J., Rebowski, G., Boczkowska, M., Heuckeroth, R. O. and Dominguez, R. (2022). A solution to the long-standing problem of actin expression and purification. *Proc. Natl. Acad. Sci. USA* 119, e2209150119. 36197995 10.1073/pnas.2209150119 PMC9565351

[JCS260540C10] Chesarone, M. A., DuPage, A. G. and Goode, B. L. (2010). Unleashing formins to remodel the actin and microtubule cytoskeletons. *Nat. Rev. Mol. Cell Biol.* 11, 62-74. 10.1038/nrm2816 19997130

[JCS260540C11] Chin, S. M., Hatano, T., Sivashanmugam, L., Suchenko, A., Kashina, A. S., Balasubramanian, M. K. and Jansen, S. (2022). N-terminal acetylation and arginylation of actin determines the architecture and assembly rate of linear and branched actin networks. *J. Biol. Chem.* 298, 102518. 10.1016/j.jbc.2022.102518 36152749 PMC9597890

[JCS260540C12] Cook, R. K., Sheff, D. R. and Rubenstein, P. A. (1991). Unusual metabolism of the yeast actin amino terminus. *J. Biol. Chem.* 266, 16825-16833. 10.1016/S0021-9258(18)55376-X 1885608

[JCS260540C13] Cook, R. K., Blake, W. T. and Rubenstein, P. A. (1992). Removal of the amino-terminal acidic residues of yeast actin. Studies in vitro and in vivo. *J. Biol. Chem.* 267, 9430-9436. 10.1016/S0021-9258(19)50441-0 1349604

[JCS260540C14] De La Cruz, E. M. (2005). Cofilin binding to muscle and non-muscle actin filaments: isoform-dependent cooperative interactions. *J. Mol. Biol.* 346, 557-564. 10.1016/j.jmb.2004.11.065 15670604

[JCS260540C15] Drazic, A., Timmerman, E., Kajan, U., Marie, M., Varland, S., Impens, F., Gevaert, K. and Arnesen, T. (2022). The final maturation state of β-actin involves N-terminal acetylation by NAA80, not N-terminal arginylation by ATE1. *J. Mol. Biol.* 434, 167397. 10.1016/j.jmb.2021.167397 34896361 PMC7613935

[JCS260540C16] Ferron, F., Rebowski, G., Lee, S. H. and Dominguez, R. (2007). Structural basis for the recruitment of profilin-actin complexes during filament elongation by Ena/VASP. *EMBO J.* 26, 4597-4606. 10.1038/sj.emboj.7601874 17914456 PMC2063483

[JCS260540C17] Geissler, S., Siegers, K. and Schiebel, E. (1998). A novel protein complex promoting formation of functional alpha- and gamma-tubulin. *EMBO J.* 17, 952-966. 10.1093/emboj/17.4.952 9463374 PMC1170445

[JCS260540C18] Grantham, J. (2020). The molecular chaperone CCT/TRiC: an essential component of proteostasis and a potential modulator of protein aggregation. *Front. Genet.* 11, 172. 10.3389/fgene.2020.00172 32265978 PMC7096549

[JCS260540C72] Haarer, B., Viggiano, S., Hibbs, M. A., Troyanskaya, O. G. and Amberg, D. C. (2007). Modeling complex genetic interactions in a simple eukaryotic genome: actin displays a rich spectrum of complex haploinsufficiencies. *Genes Dev.* 21, 148-159. 10.1101/gad.1477507 17167106 PMC1770898

[JCS260540C19] Hatano, T., Alioto, S., Roscioli, E., Palani, S., Clarke, S. T., Kamnev, A., Hernandez-Fernaud, J. R., Sivashanmugam, L., Chapa, Y. L. B., Jones, A. M. E. et al. (2018). Rapid production of pure recombinant actin isoforms in Pichia pastoris. *J. Cell Sci.* 131, jcs213827. 10.1242/jcs.213827 29535210 PMC5976186

[JCS260540C20] Hatano, T., Sivashanmugam, L., Suchenko, A., Hussain, H. and Balasubramanian, M. K. (2020). Pick-ya actin - a method to purify actin isoforms with bespoke key post-translational modifications. *J. Cell Sci.* 133, jcs241406. 10.1242/jcs.241406 31964701 PMC7615240

[JCS260540C21] Henty-Ridilla, J. L. (2022). Visualizing actin and microtubule coupling dynamics in vitro by total internal reflection fluorescence (TIRF) microscopy. *J. Vis. Exp.* 185, e64074. 10.3791/64074 PMC10132125 35938818

[JCS260540C22] Hertzog, M. and Carlier, M. (2005). Functional characterization of proteins regulating actin assembly. *Curr. Protoc. Cell Biol.* 26, 13.6.1-13.6.23. 10.1002/0471143030.cb1306s26 18228461

[JCS260540C23] Higaki, T., Kutsuna, N., Sano, T., Kondo, N. and Hasezawa, S. (2010). Quantification and cluster analysis of actin cytoskeletal structures in plant cells: role of actin bundling in stomatal movement during diurnal cycles in *Arabidopsis* guard cells. *Plant J.* 61, 156-165. 10.1111/j.1365-313X.2009.04032.x 20092030

[JCS260540C73] Hill, J. E., Myers, A. M., Koerner, T. J. and Tzagoloff, A. (1986). Yeast/E. coli shuttle vectors with multiple unique restriction sites. *Yeast* 2, 163-167. 10.1002/yea.320020304 3333305

[JCS260540C24] Hundt, N., Preller, M., Swolski, O., Ang, A. M., Mannherz, H. G., Manstein, D. J. and Muller, M. (2014). Molecular mechanisms of disease-related human beta-actin mutations p.R183W and p.E364K. *FEBS J.* 281, 5279-5291. 10.1111/febs.13068 25255767

[JCS260540C25] Jansen, S., Collins, A., Yang, C., Rebowski, G., Svitkina, T. and Dominguez, R. (2011). Mechanism of actin filament bundling by fascin. *J. Biol. Chem.* 286, 30087-30096. 21685497 10.1074/jbc.M111.251439 PMC3191048

[JCS260540C26] Kalhor, H. R., Niewmierzycka, A., Faull, K. F., Yao, X., Grade, S., Clarke, S. and Rubenstein, P. A. (1999). A Highly Conserved 3-Methylhistidine Modification is absent in yeast actin. *Arch. Biochem. Biophys.* 370, 105-111. 10.1006/abbi.1999.1370 10496983

[JCS260540C27] Karakozova, M., Kozak, M., Wong, C. C., Bailey, A. O., Yates, J. R., III, Mogilner, A., Zebroski, H. and Kashina, A. (2006). Arginylation of beta-actin regulates actin cytoskeleton and cell motility. *Science* 313, 192-196. 10.1126/science.1129344 16794040

[JCS260540C28] Karlsson, R. (1988). Expression of chicken beta-actin in *Saccharomyces cerevisiae*. *Gene* 68, 249-257. 10.1016/0378-1119(88)90027-3 3065145

[JCS260540C29] Karlsson, R., Aspenstrom, P. and Bystrom, A. S. (1991). A chicken beta-actin gene can complement a disruption of the *Saccharomyces cerevisiae* ACT1 gene. *Mol. Cell. Biol.* 11, 213-217. 1986221 10.1128/mcb.11.1.213-217.1991 PMC359611

[JCS260540C30] Khurana, P., Henty, J. L., Huang, S., Staiger, A. M., Blanchoin, L. and Staiger, C. J. (2010). *Arabidopsis VILLIN1* and *VILLIN3* have overlapping and distinct activities in actin bundle formation and turnover. *Plant Cell* 22, 2727-2748. 10.1105/tpc.110.076240 20807878 PMC2947172

[JCS260540C31] Kijima, S. T., Hirose, K., Kong, S. G., Wada, M. and Uyeda, T. Q. (2016). Distinct biochemical properties of *Arabidopsis thaliana* actin isoforms. *Plant Cell Physiol.* 57, 46-56. 10.1093/pcp/pcv176 26578694

[JCS260540C32] Kinosian, H. J., Selden, L. A., Gershman, L. C. and Estes, J. E. (2000). Interdependence of profilin, cation, and nucleotide binding to vertebrate non-muscle actin. *Biochemistry* 39, 13176-13188. 10.1021/bi001520+ 11052670

[JCS260540C33] Kuhn, J. R. and Pollard, T. D. (2005). Real-time measurements of actin filament polymerization by total internal reflection fluorescence microscopy. *Biophys. J.* 88, 1387-1402. 10.1529/biophysj.104.047399 15556992 PMC1305141

[JCS260540C34] Kumar, A., Birnbaum, M. D., Patel, D. M., Morgan, W. M., Singh, J., Barrientos, A. and Zhang, F. (2016). Posttranslational arginylation enzyme Ate1 affects DNA mutagenesis by regulating stress response. *Cell Death Dis.* 7, e2378. 10.1038/cddis.2016.284 27685622 PMC5059882

[JCS260540C35] Li, F. and Higgs, H. N. (2003). The mouse Formin mDia1 is a potent actin nucleation factor regulated by autoinhibition. *Curr. Biol.* 13, 1335-1340. 10.1016/S0960-9822(03)00540-2 12906795

[JCS260540C36] Li, M.-M., Nilsen, A., Shi, Y., Fusser, M., Ding, Y.-H., Fu, Y., Liu, B., Niu, Y., Wu, Y.-S., Huang, C.-M. et al. (2013). ALKBH4-dependent demethylation of actin regulates actomyosin dynamics. *Nat. Commun.* 4, 1832. 10.1038/ncomms2863 23673617 PMC3674258

[JCS260540C37] Liu, X., Pimm, M. L., Haarer, B., Brawner, A. T. and Henty-Ridilla, J. L. (2022). Biochemical characterization of actin assembly mechanisms with ALS-associated profilin variants. *Eur. J. Cell Biol.* 101, 151212. 10.1016/j.ejcb.2022.151212 35248815 PMC10163920

[JCS260540C38] Lu, H., Fagnant, P. M., Bookwalter, C. S., Joel, P. and Trybus, K. M. (2015). Vascular disease-causing mutation R258C in ACTA2 disrupts actin dynamics and interaction with myosin. *Proc. Natl. Acad. Sci. USA* 112, E4168-E4177. 26153420 10.1073/pnas.1507587112 PMC4534267

[JCS260540C39] McKane, M., Wen, K.-K., Boldogh, I. R., Ramcharan, S., Pon, L. A. and Rubenstein, P. A. (2005). A mammalian actin substitution in yeast actin (H372R) causes a suppressible mitochondria/vacuole phenotype. *J. Biol. Chem.* 280, 36494-36501. 10.1074/jbc.M506970200 16118223

[JCS260540C40] McKane, M., Wen, K.-K., Meyer, A. and Rubenstein, P. A. (2006). Effect of the substitution of muscle actin-specific subdomain 1 and 2 residues in yeast actin on actin function. *J. Biol. Chem.* 281, 29916-29928. 10.1074/jbc.M602251200 16882670

[JCS260540C41] Millán-Zambrano, G. and Chavez, S. (2014). Nuclear functions of prefoldin. *Open Biol.* 4, 140085. 10.1098/rsob.140085 25008233 PMC4118604

[JCS260540C42] Moradi, M., Sivadasan, R., Saal, L., Luningschror, P., Dombert, B., Rathod, R. J., Dieterich, D. C., Blum, R. and Sendtner, M. (2017). Differential roles of alpha-, beta-, and gamma-actin in axon growth and collateral branch formation in motoneurons. *J. Cell Biol.* 216, 793-814. 28246119 10.1083/jcb.201604117 PMC5346967

[JCS260540C43] Mu, A., Fung, T. S., Francomacaro, L. M., Huynh, T., Kotila, T., Svindrych, Z. and Higgs, H. N. (2020). Regulation of INF2-mediated actin polymerization through site-specific lysine acetylation of actin itself. *Proc. Natl. Acad. Sci. USA* 117, 439-447. 31871199 10.1073/pnas.1914072117 PMC6955303

[JCS260540C44] Müller, M., Mazur, A. J., Behrmann, E., Diensthuber, R. P., Radke, M. B., Qu, Z., Littwitz, C., Raunser, S., Schoenenberger, C. A., Manstein, D. J. et al. (2012). Functional characterization of the human alpha-cardiac actin mutations Y166C and M305L involved in hypertrophic cardiomyopathy. *Cell. Mol. Life Sci.* 69, 3457-3479. 10.1007/s00018-012-1030-5 22643837 PMC11115188

[JCS260540C45] Müller, M., Diensthuber, R. P., Chizhov, I., Claus, P., Heissler, S. M., Preller, M., Taft, M. H. and Manstein, D. J. (2013). Distinct functional interactions between actin isoforms and nonsarcomeric myosins. *PLoS ONE* 8, e70636. 10.1371/journal.pone.0070636 23923011 PMC3724804

[JCS260540C46] Namba, Y., Ito, M., Zu, Y., Shigesada, K. and Maruyama, K. (1992). Human T cell L-plastin bundles actin filaments in a calcium-dependent manner. *J. Biochem.* 112, 503-507. 1491005 10.1093/oxfordjournals.jbchem.a123929

[JCS260540C47] Nguyen, K. T., Kim, J.-M., Park, S.-E. and Hwang, C.-S. (2019). N-terminal methionine excision of proteins creates tertiary destabilizing N-degrons of the Arg/N-end rule pathway. *J. Biol. Chem.* 294, 4464-4476. 10.1074/jbc.RA118.006913 30674553 PMC6433082

[JCS260540C48] Noguchi, T. Q., Kanzaki, N., Ueno, H., Hirose, K. and Uyeda, T. Q. (2007). A novel system for expressing toxic actin mutants in Dictyostelium and purification and characterization of a dominant lethal yeast actin mutant. *J. Biol. Chem.* 282, 27721-27727. 10.1074/jbc.M703165200 17656358

[JCS260540C49] Ohki, T., Ohno, C., Oyama, K., Mikhailenko, S. V. and Ishiwata, S. (2009). Purification of cytoplasmic actin by affinity chromatography using the C-terminal half of gelsolin. *Biochem. Biophys. Res. Commun.* 383, 146-150. 10.1016/j.bbrc.2009.03.144 19344694

[JCS260540C50] Parker, F., Baboolal, T. G. and Peckham, M. (2020). Actin mutations and their role in disease. *Int. J. Mol. Sci.* 21, 3371. 10.3390/ijms21093371 32397632 PMC7247010

[JCS260540C51] Patel, A. A., Oztug Durer, Z. A., van Loon, A. P., Bremer, K. V. and Quinlan, M. E. (2018). Drosophila and human FHOD family formin proteins nucleate actin filaments. *J. Biol. Chem.* 293, 532-540. 10.1074/jbc.M117.800888 29127202 PMC5767859

[JCS260540C52] Perrin, B. J. and Ervasti, J. M. (2010). The actin gene family: function follows isoform. *Cytoskeleton* 67, 630-634. 10.1002/cm.20475 20737541 PMC2949686

[JCS260540C53] Pimm, M. L., Hotaling, J. and Henty-Ridilla, J. L. (2020b). Profilin choreographs actin and microtubules in cells and cancer. *Int. Rev. Cell Mol. Biol.* 355, 155-204. 10.1016/bs.ircmb.2020.05.005 32859370 PMC7461721

[JCS260540C54] Pimm, M. L., Liu, X., Tuli, F., Heritz, J., Lojko, A. and Henty-Ridilla, J. L. (2022). Visualizing molecules of functional human profilin. *eLife* 11, e76485. 10.7554/eLife.76485 35666129 PMC9249392

[JCS260540C55] Rappsilber, J., Ishihama, Y. and Mann, M. (2003). Stop and go extraction tips for matrix-assisted laser desorption/ionization, nanoelectrospray, and LC/MS sample pretreatment in proteomics. *Anal. Chem.* 75, 663-670. 10.1021/ac026117i 12585499

[JCS260540C56] Rutkevich, L. A., Teal, D. J. and Dawson, J. F. (2006). Expression of actin mutants to study their roles in cardiomyopathy. *Can. J. Physiol. Pharmacol.* 84, 111-119. 16845895 10.1139/Y05-140

[JCS260540C57] Schafer, D. A., Jennings, P. B. and Cooper, J. A. (1998). Rapid and efficient purification of actin from nonmuscle sources. *Cell Motil. Cytoskeleton* 39, 166-171. 10.1002/(SICI)1097-0169(1998)39:2<166::AID-CM7>3.0.CO;2-4 9484958 PMC2362386

[JCS260540C58] Schindelin, J., Arganda-Carreras, I., Frise, E., Kaynig, V., Longair, M., Pietzsch, T., Preibisch, S., Rueden, C., Saalfeld, S., Schmid, B. et al. (2012). Fiji: an open-source platform for biological-image analysis. *Nat. Methods* 9, 676-682. 10.1038/nmeth.2019 22743772 PMC3855844

[JCS260540C59] Skruber, K., Read, T. A. and Vitriol, E. A. (2018). Reconsidering an active role for G-actin in cytoskeletal regulation. *J. Cell Sci.* 131, jcs203760. 10.1242/jcs.203760 29321224 PMC5818056

[JCS260540C60] Skruber, K., Warp, P. V., Shklyarov, R., Thomas, J. D., Swanson, M. S., Henty-Ridilla, J. L., Read, T. A. and Vitriol, E. A. (2020). Arp2/3 and Mena/VASP require Profilin 1 for actin network assembly at the leading edge. *Curr. Biol.* 30, 2651-2664.e2655. 32470361 10.1016/j.cub.2020.04.085 PMC7375932

[JCS260540C61] Steger, C. (1998). An unbiased detector of curvilinear structures. *IEEE Trans. Pattern Anal. Mach. Intell.* 20, 113-125. 10.1109/34.659930

[JCS260540C62] Tamura, M., Ito, K., Kunihiro, S., Yamasaki, C. and Haragauchi, M. (2011). Production of human beta-actin and a mutant using a bacterial expression system with a cold shock vector. *Protein Expr. Purif.* 78, 1-5. 10.1016/j.pep.2010.09.007 20851184

[JCS260540C63] Terman, J. R. and Kashina, A. (2013). Post-translational modification and regulation of actin. *Curr. Opin. Cell Biol.* 25, 30-38. 10.1016/j.ceb.2012.10.009 23195437 PMC3578039

[JCS260540C64] Valencia, D. A. and Quinlan, M. E. (2021). Formins. *Curr. Biol.* 31, R517-R522. 10.1016/j.cub.2021.02.047 34033783

[JCS260540C65] Valpuesta, J. M., Martin-Benito, J., Gomez-Puertas, P., Carrascosa, J. L. and Willison, K. R. (2002). Structure and function of a protein folding machine: the eukaryotic cytosolic chaperonin CCT. *FEBS Lett.* 529, 11-16. 10.1016/S0014-5793(02)03180-0 12354605

[JCS260540C66] Varland, S., Vandekerckhove, J. and Drazic, A. (2019). Actin post-translational modifications: the cinderella of cytoskeletal control. *Trends Biochem. Sci.* 44, 502-516. 10.1016/j.tibs.2018.11.010 30611609

[JCS260540C67] von der Ecken, J., Heissler, S. M., Pathan-Chhatbar, S., Manstein, D. J. and Raunser, S. (2016). Cryo-EM structure of a human cytoplasmic actomyosin complex at near-atomic resolution. *Nature* 534, 724-728. 10.1038/nature18295 27324845

[JCS260540C68] Wagner, T., M. Hiner, and Xraynaud, . (2017). Thorstenwagner/Ij-Ridgedetection: ridge detection 1.4.0. Zenodo.

[JCS260540C69] Wilkinson, A. W., Diep, J., Dai, S., Liu, S., Ooi, Y. S., Song, D., Li, T.-M., Horton, J. R., Zhang, X., Liu, C. et al. (2019). SETD3 is an actin histidine methyltransferase that prevents primary dystocia. *Nature* 565, 372-376. 10.1038/s41586-018-0821-8 30626964 PMC6511263

[JCS260540C70] Xu, T., Wong, C. C. L., Kashina, A. and Yates, J. R. (2009). Identification of N-terminally arginylated proteins and peptides by mass spectrometry. *Nat. Protoc.* 4, 325-332. 10.1038/nprot.2008.248 19229197 PMC2683362

[JCS260540C71] Yamashiro, S., Gokhin, D. S., Sui, Z., Bergeron, S. E., Rubenstein, P. A. and Fowler, V. M. (2014). Differential actin-regulatory activities of Tropomodulin1 and Tropomodulin3 with diverse tropomyosin and actin isoforms. *J. Biol. Chem.* 289, 11616-11629. 10.1074/jbc.M114.555128 24644292 PMC4002072

