# Supplementary material for: Purification of human β- and γ-actin from budding yeast
Source: J Cell Sci. 2023 May 9;136(9):jcs260540. doi: 10.1242/jcs.260540 (PMC10184827; doi:10.1242/jcs.260540)
Supplement: Supplementary information [file joces-136-260540-s1.pdf]

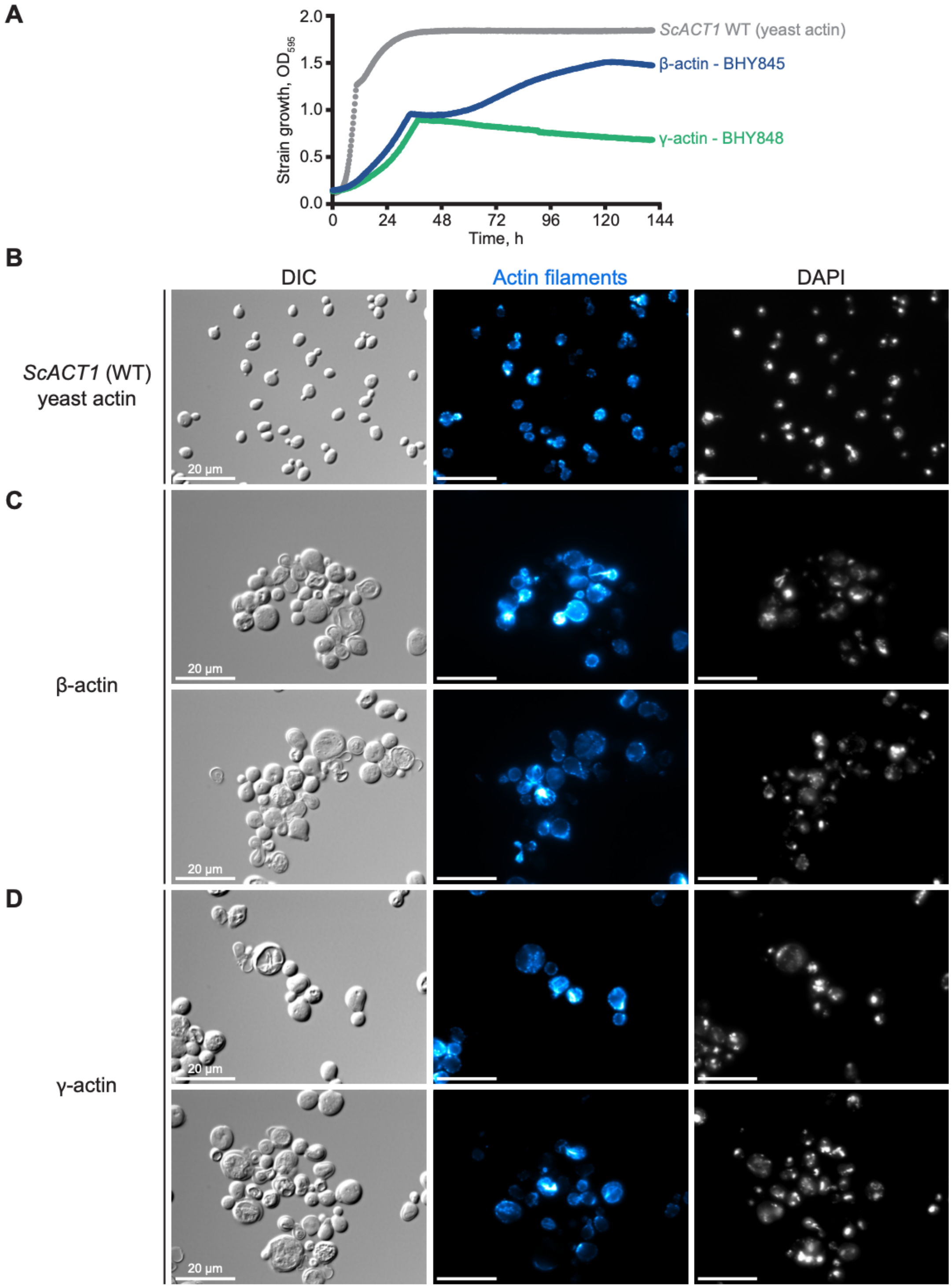

Fig. S1. Growth and appearance of yeast expressing β- or γ-actin isoforms. (A) Growth curves of yeast strains expressing yeast actin (ACT1; grey), human β-actin (blue), or human γ-actin (green). **(B-D)** Representative DIC and epifluorescence images of yeast expressing (B) yeast actin, (C) human β-actin, or (D) human γ-actin. Left, DIC; middle, rhodamine-phalloidin; right, DAPI. Scale bars, 20 μm.

Journal of Cell Science • Supplementary information

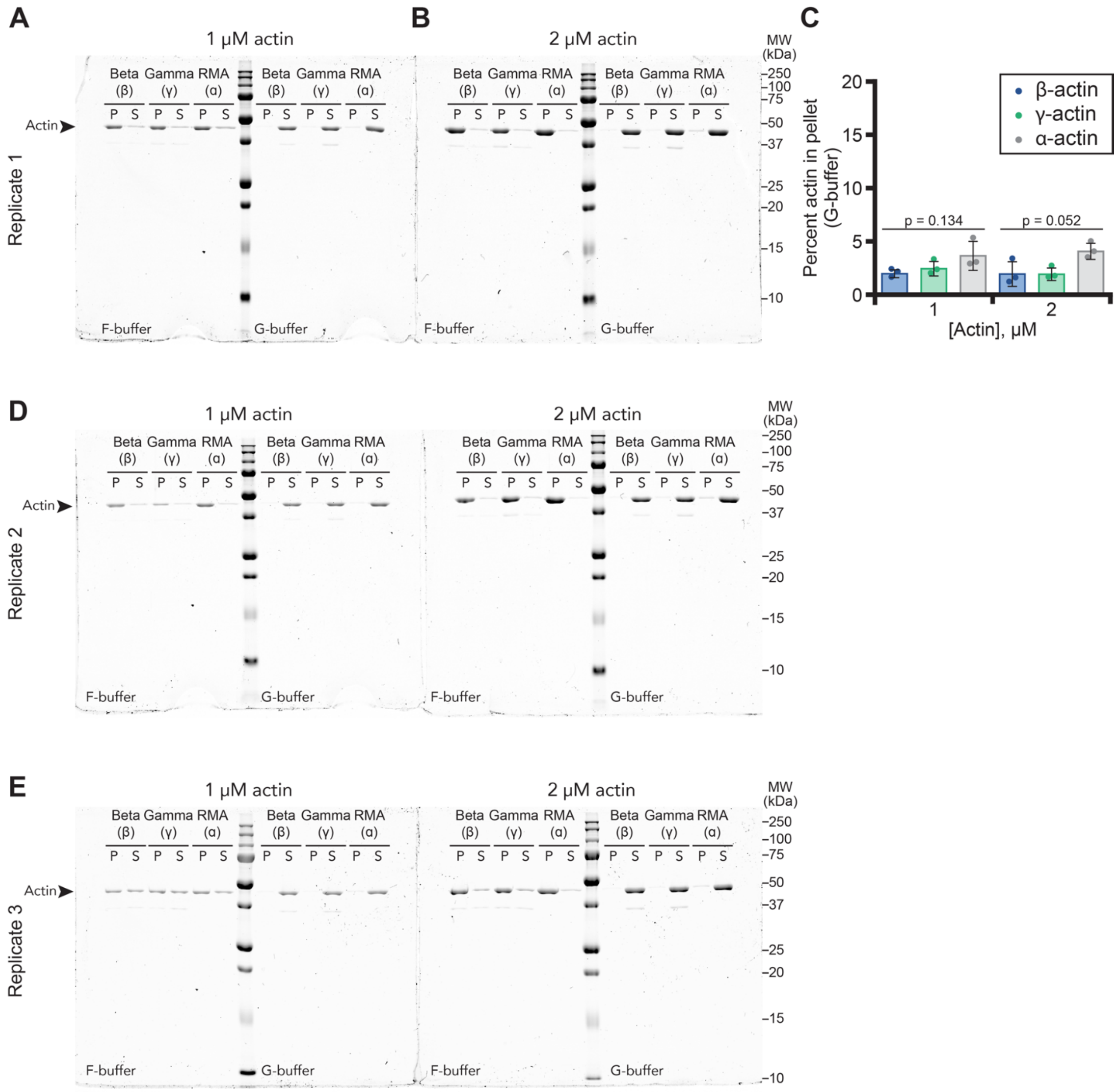

Fig. S2. Full gels associated with analysis in Figure 2C. (A) Individual gel displaying (P; pellet) or (S; supernatant) fractions from pelleting assays performed with 1 μM actin filaments (F-buffer) or actin monomers (G-buffer). **(B)** Gel as in (A) with 2 μM actin filaments or monomers. Gels shown in A and B are the full gels from Figure 2A and B. **(C)** Quantification by band densitometry of pellet samples from (A-B). Statistics: ANOVA; treatments were not significantly different. **(D-E)** Gels from additional replicates for analysis in (C).

Journal of Cell Science • Supplementary information

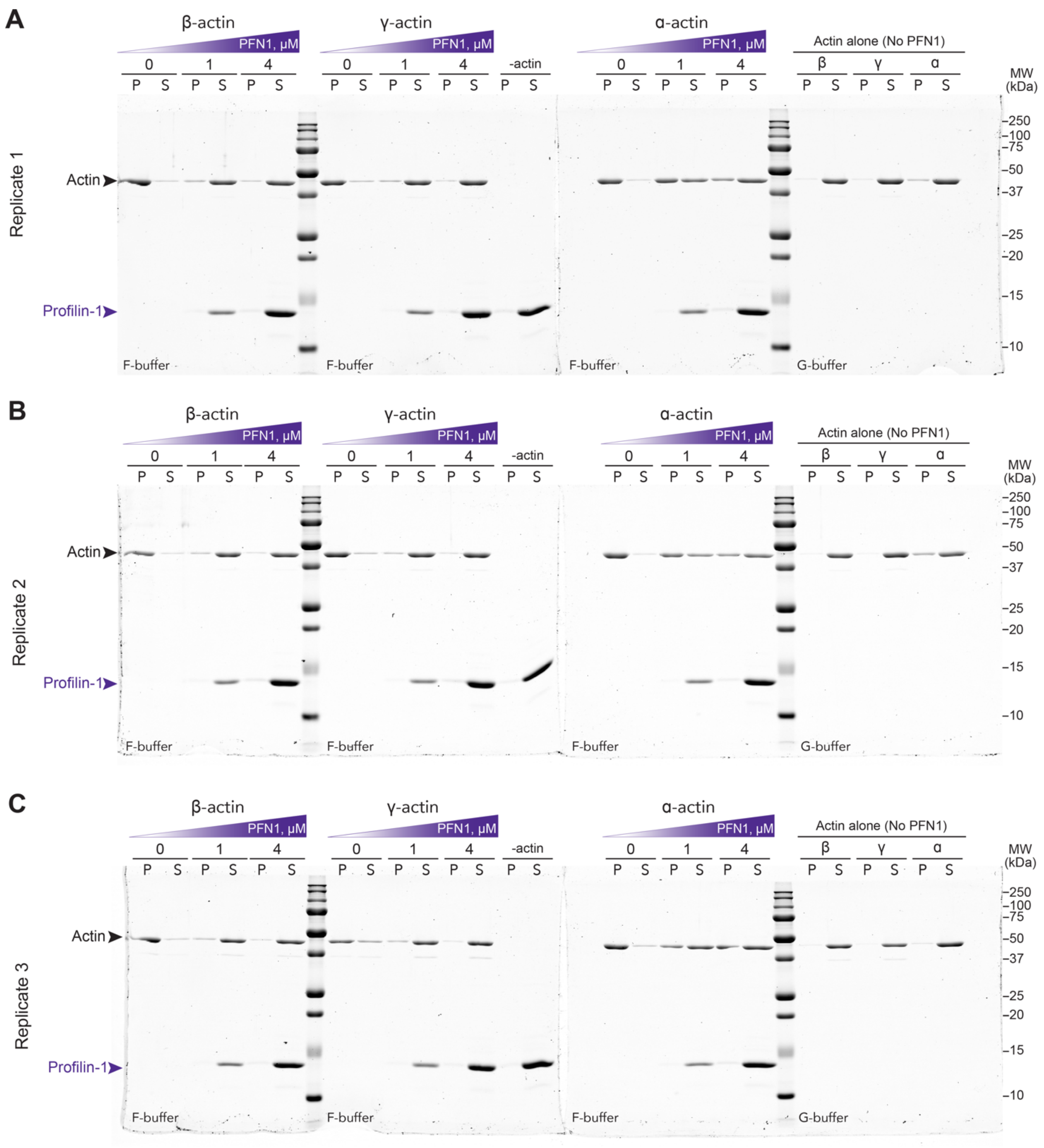

Fig. S3. Full gels associated with analysis in Figure 4C. (A-C) Full scans of Individual gels displaying (P; pellet) or (S; supernatant) fractions from pelleting assays performed with 2 μM actin filaments (F-actin) and varying amounts of profilin-1 (PFN1; purple arrows). Actin monomer controls (G-buffer) lacking PFN1 are also shown. Panel (A) shows the gels used in Figure 4B.

Journal of Cell Science • Supplementary information

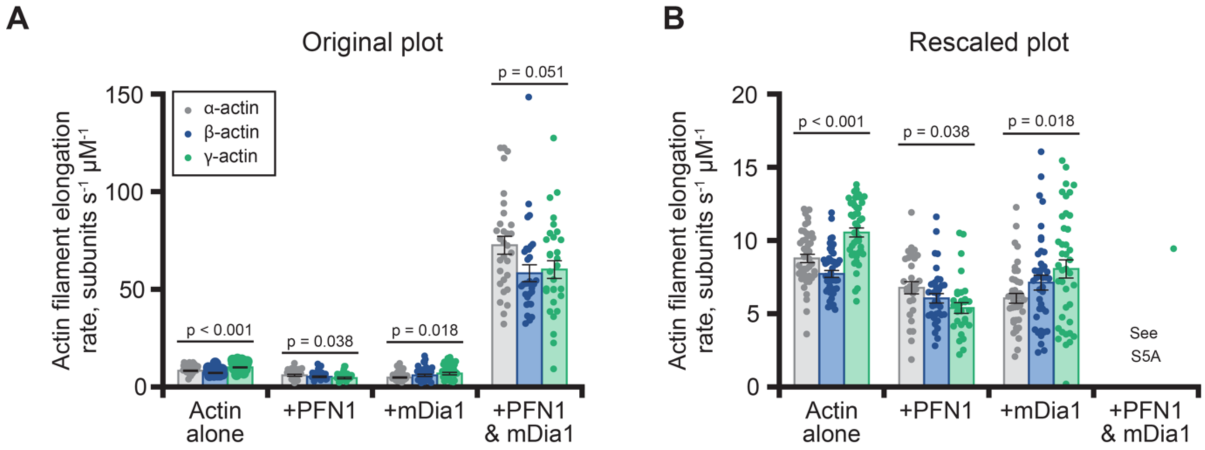

C

Multiple statistical comparisons

Condition	Comparison	p-value	n1	n2
Actin alone	α-actin to β-actin	0.0244	45	45
	α-actin to γ-actin	<0.0001	45	45
	β-actin to γ-actin	<0.0001	45	45
+PFN1	α-actin to β-actin	0.3177	30	35
	α-actin to γ-actin	0.0292	30	30
	β-actin to γ-actin	0.4325	35	30
+mDia1	α-actin to β-actin	0.2722	40	40
	α-actin to γ-actin	0.0128	40	40
	β-actin to γ-actin	0.3773	40	40
+PFN1 & mDia1	α-actin to β-actin	0.0640	30	30
	α-actin to γ-actin	0.1232	30	30
	β-actin to γ-actin	0.9518	30	30

Fig. S4. Comparison of 4F and rescaled version to view slower elongation rates. (A) Original plot of actin filament elongation rates from Figure 4F. **(B)** Data from Figure 4F or panel (A) rescaled to make the distributions of slower elongation rates more visible. Statistics: ANOVA comparison of all treatments under the line. **(C)** Table displaying additional individual statistical comparisons.

Journal of Cell Science • Supplementary information

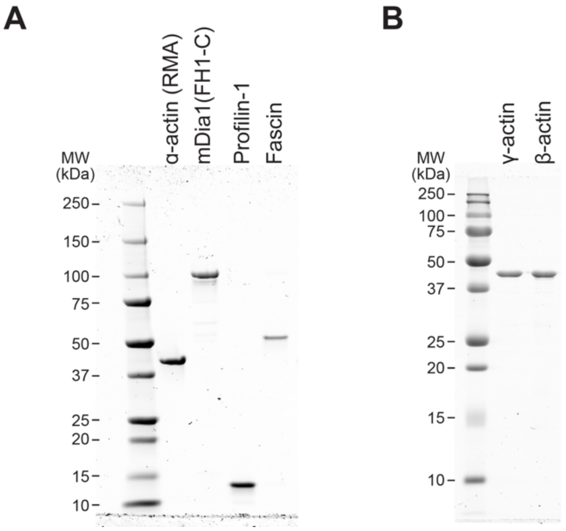

Fig. S5. Purity of proteins used in TIRF assays.

(A-B) Coomassie-stained gels showing the purity of proteins used in this study, as follows: **(A)** 360 ng α-actin (RMA), 375 ng mDia1(FH1-C), 165 ng Profilin-1, 280 ng Fascin, **(B)** 390 ng β-actin, and 310 ng γ-actin.

Journal of Cell Science • Supplementary information

Table S1. Mass Spectrometry-based actin secondary modifications.

Modification[1]	α-actin (RMA)[2]	β-actin	γ-actin
Acetylation	D1[2], K50, K68, K215, K238, K284, K291, K315	M1, K18, K50	M1, K18, K238
Methylation	K61, K68, H73, K84, H87, H88, H101, N225, N252, K291, N296, N 297, H371	H40, H87, H88, H101, N128, H161, N252, K291, N296	N12, K18, H87, H88, H101, H161, N252, K291, N296, H371
Arginylation	H40, D51, W86, F90, A97, K18, T120, T148, L216, C217, V219, A220, C285, K291, D292, L293, E316, Q360	D3, D4, V10, D51, W86, A97, P98, T120, A135, G150, C217, A220, D292, L299, I329, Q360	M1, E4, I10, D51, S52, Y69, D80, W86, A97, P98, T120, A135, G150, T160, C217, A220, D292, I329, Q360

[1]Data are presented for peptide mass searches for N-terminal or lysine acetylation, methylation, and arginylation.
[2]Numbering of α-actin (RMA) starts at the third amino acid due to the N-terminal truncation of Met-Cys. Thus, Asp3 is listed as D1.

Table S2. N-terminal peptide modifications.

Modification	α-actin (RMA)[1]	β-actin	γ-actin
Acetylation	D1[1] (4/5)	M1 (11/12)<br>K18 (7/12)	M1 (3/4)<br>K18 (1/4)
Arginylation[2]	None detected	D3 (5/9)<br>D4 (3/4)	E4 (1/3)

[1]Numbering of α-actin (RMA) starts at third amino acid due to N-terminal truncation of Met-Cys.
[2]Arginylation detected as N-terminal modification to truncated peptides.

Table S3. Peptide matches for beta, gamma, and RMA compared to their respective amino acid sequences. Included tabs are the results from searches performed for each actin allowing for: (1) N-acetylation, lysine acetylation, N-arginylation, methionine oxidation, and cysteine carbamidomethyl modification; (2) methylation of lysine, histidine, or asparagine (designated CH3). Full-length and truncated actins included in the peptide searches are included in the “modified actins” tab.

Click here to download Table S3

Journal of Cell Science • Supplementary information

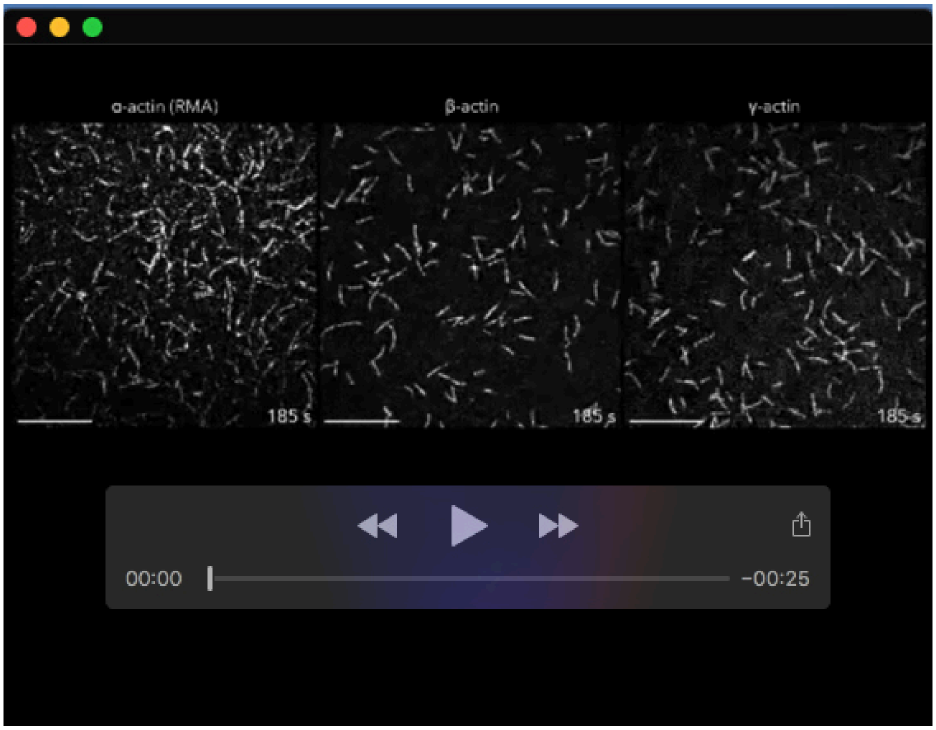

Movie 1. β- and γ-actin form actin filament polymers. TIRF movies comparing the polymerization of actin isoforms from reactions containing: 1 μM actin of each isoform (10% Alexa- 488 RMA). Scale bar, 20 μm. Playback, 10 fps.

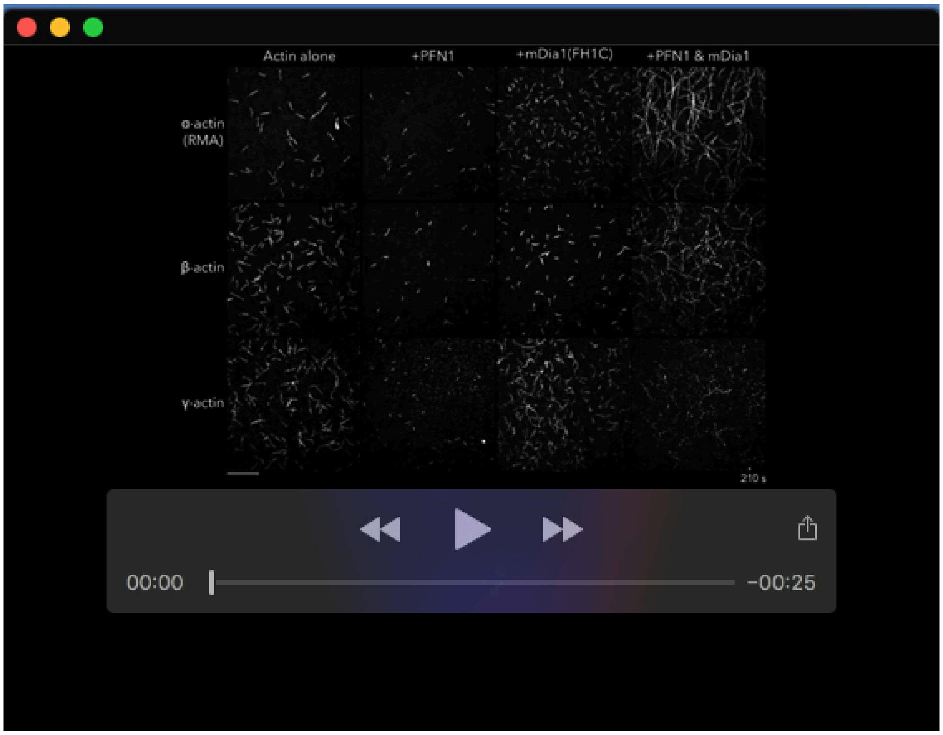

Movie 2. The formin mDia1 can nucleate and rapidly elongate β- and γ-actin filaments. TIRF movies of reactions containing labeled combinations of 1 μM α-actin, β-actin, or γ-actin (10% Alexa-488 RMA),10 nM mDia1(FH1-C), and 2 μM profilin-1. Scale bar, 20 μm. Playback, 10 fps.

Journal of Cell Science • Supplementary information
