# Supplementary material for: Purification of human β- and γ-actin from budding yeast
Source: J Cell Sci. 2023 May 9;136(9):jcs260540. doi: 10.1242/jcs.260540 (PMC10184827; doi:10.1242/jcs.260540)
Supplement: Supplementary file 1 [file joces-136-260540_review_history.pdf]

Purification of human β- and γ-actin from budding yeast

Brian K. Haarer, Morgan L. Pimm, Ebbing P. de Jong, David C. Amberg and Jessica L. Henty-Ridilla
DOI: 10.1242/jcs.260540

Editor: Michael Way

Review timeline

Original submission:	17 August 2022
Editorial decision:	7 November 2022
First revision received:	21 February 2023
Editorial decision:	30 March 2023
Second revision received:	4 April 2023
Accepted:	4 April 2023

Original submission

First decision letter

MS ID#: JOCES/2022/260540

MS TITLE: Purification of Human ß- and γ Actin from Budding Yeast

AUTHORS: Brian K Haarer, Morgan L Pimm, Ebbing P deJong, Jessica L Henty-Ridilla, and David C Amberg
ARTICLE TYPE: Tools and Resources

We have now reached a decision on the above manuscript but I must apologise for the length of time it has taken. We have been chasing the third reviewer for some time, however, unfortunately we have not managed to obtain their review.

To see the reviewers' reports and a copy of this decision letter, please go to: https://submit-jcs.biologists.organd click on the 'Manuscripts with Decisions' queue in the Author Area. (Corresponding author only has access to reviews.)

As you will see, the reviewers were positive but are quite polarised in their comments, which is why we waited for the third reviewer but given the time I think it was better make a decision. As you will see reviewer 1 raises a number criticisms and suggests providing more biochemical repeats or to focus on the the actual purification method and the yeast cells (a point also raised by reviewer 2). Reviewer 2 also wonders if you can improve your system by expressing NAA80 and /or SETD3. I'm sure it might help but I think that is beyond the scope of the current paper as it will require a substantial amount of additional work. I leave it up to you which route you feel you want to go and you might even want to do both but I would be happy with either. We would then return it to the reviewers.

Please ensure that you clearly highlight all changes made in the revised manuscript. Please avoid using 'Tracked changes' in Word files as these are lost in PDF conversion.

I should be grateful if you would also provide a point-by-point response detailing how you have dealt with the points raised by the reviewers in the 'Response to Reviewers' box. Please attend to

© 2023. Published by The Company of Biologists under the terms of the Creative Commons Attribution License (https://creativecommons.org/licenses/by/4.0/).

all of the reviewers' comments. If you do not agree with any of their criticisms or suggestions please explain clearly why this is so.

Reviewer 1

Advance summary and potential significance to field

This paper reports a method to produce recombinant beta and gamma-1 actin in yeast (Saccharomyces cerevisiae). Yeast expresses a single actin isoform, which in this study is substituted by either human beta or gamma-1 actin as the sole actin in the cell. This simplifies purification and is important because it resolves the issue of contamination with endogenous actin of the expressing cells that affects most existing actin expression methods. Another advantage of this method is that it does not require post-purification steps, such as affinity tag removal. This point could be further emphasized by pointing out that chymotrypsin, a protease commonly used to remove affinity tags from recombinant actin, cleaves actin at several sites. In principle, I would like to support publication of this paper in Journal of Cell Science, but first several issues need to be addressed.

Comments for the author

While this work has the potential to be interesting and impactful, several issues are noted, specifically the lack of adequate replicates, quantifications, statistical analyses, and controls. As a result, the limited biochemical analysis of expressed actin isoforms shown harms the paper instead of enhancing its impact. There are two ways around this problem. The paper could simply focus on the expression-purification method with a more thorough analysis of the yeast strains expressing these isoforms. Alternatively, the authors could perform more replicates, quantifications, and statistical analyses. Another problem is with post-translational modification, which is poorly addressed while being a key factor for actin function. These issues are specifically developed below.

Major concerns:

(1) There are several issues with post-translational modification and its treatment in this paper. One of the disadvantages of yeast as an expression system for actin is that it fails to add important post-translational modifications known to impact actin's function in mammals. The authors need to state this clearly, and specifically in the following two instances: On page 4, "While actin purified by this approach may not undergo standard N-terminal processing…" should be "While actin purified by this approach does not undergo standard human N-terminal processing …". On page 12, "A potential drawback of our system is that yeast-produced actin may lack specific…" should be "… yeast-produced actin lacks specific…".

One study (PMID: 29581253) reported that the mere absence of actin's N-terminal acetyl group can lead to substantial effects on filament elongation rate for actin alone and by the formin mDia1. His73 methylation on the other hand reduces the nucleotide-exchange rate on actin and accelerates filament assembly (PMID: 30626964). Other PTMs like phosphorylation, Lys-acetylation also affect actin's function and interactions with ABPs, but are not mentioned here. If PTMs are different for beta and gamma-1 actin (as reported here for example for N-terminal acetylation/arginylation), this raises questions about the relevance of these experiments and reported difference between these isoforms.

(2) Mass spectrometry analysis reported should include representative MS2 spectra (including Y and B ions), a listing of all the observed peptides, and proper quantification of PTM peptides vs. total # of observed peptides. The proteomics data should be deposited with an appropriate database (as customary). The reported arginylation of beta actin on the 3rd residue is specifically problematic because it cannot be reliable distinguished in mass from N-terminal acetylation on the 2nd residue.

(3) Many experiments lack replicates, quantification, statistical comparisons, and the alpha skeletal actin control. Specific comments per figures/experiments:

© 2023. Published by The Company of Biologists under the terms of the Creative Commons Attribution License (https://creativecommons.org/licenses/by/4.0/).

a. Figure 1A: The coloring for acetylation and arginylation does not match what is reported in the discussion.
b. Figure 1B&C: There should be some actin in the supernatant after polymerization as polymerization reaches equilibrium, but the gels are too processed to see this.
c. Figure 1D: Pyrene-actin polymerization was conducted but not carried out to completion (plateau) and lacks proper replicates and quantification. It seems that the sole purpose of the pyrene data is to show the expressed actin polymerizes. The readers can see this in other figures (including Figure 1E&F). Additionally, it appears that gamma-1 actin may be slower than beta actin in pyrene polymerization assays, but the opposite is observed in TIRF microscopy experiments. Which experiment should we believe? Consider either removing this data or completing the experiments (at least 3 replicates that reach plateau) and quantifications of the polymerization rates.
d. Figure 2B: Please report the values ± SEM and significance for nucleation rates in the text as done for elongation rates.
e. Figure 3A: Clarify use of human cofilin-1 in methods and throughout the paper. Clarify why a yeast cofilin preparation is cited in the methods and whether it was used. Additionally, this whole experiment doesn’t make much sense. Why does so much actin pellet in the G-buffer condition? A negative control showing just cofilin-1 storage buffer w/o cofilin is missing. There is little explanation of what the gels mean in the figure legend or within the text. Could it be that cofilin-1 is crashing out causing actin to crash out and pellet with it? The rabbit alpha actin control is missing, and it is not explained why yeast actin is used instead. Replicates and gel quantifications comparing the isoforms are missing. Please correct or consider removing the cofilin experiment.
f. Figure 3C: kD values in the text don’t precisely match those in the figure.
g. Figure 3D&E: Profilin1 experiments should be quantified with at least three replicates, and the alpha skeletal actin control added.
h. Figure 3F&G and Movie: Formin mDia1 nucleates and elongates actin filaments. Therefore, TIRF quantification should be conducted for nucleation and elongation and ± profilin-1. At least three replicates and statistical comparisons should be performed for each experiment, including controls with alpha skeletal actin.
i. Figure 4B&C: The figure legend does not indicate the number of independent experiments, and it looks like in some cases n=2, which is insufficient for statistical comparison.

Minor comments:
(1) Yeast expressing alpha-actin as the sole actin did not survive. Please, cite PMID: 16118223, which made a similar observation.
(2) Most gels are overly processed and look fake.
(3) Report significant differences with brackets and asterisks (or P values) above the graphs, according to common practice.
(4) Include gels in supplementary information of all the ABPs used.
(5) Seems statistics were conducted using one-way ANOVA with Tukey post-hoc analysis, but this is not always indicated.
(6) A broad range of critical concentrations is reported (0.15 to 0.25μM), but the approach to calculate critical concentrations is not described. Either remove this information or precisely show how Cc was calculated.
(7) The authors report “high yields”, but 0.5-1 mg per L yeast culture, while adequate, cannot be considered high yield. Just listing the numbers is sufficient.

<u>Reviewer 2</u>

Advance summary and potential significance to field

The manuscript by Haarer and colleagues reports a new approach to purifying actin isoforms. Actin isoforms have been difficult to purify traditionally, although over the last decade the groups of Uyeda, Trybus, Lappalainen, and Balasubramanian have developed a number of systems (in Dicty, Pichia, sf9 cells), largely based on thymosinb4 binding and subsequent purification and chymotrypsin cleavage. The one slight complication is the potential to co purify small amounts of native actin actin.

The current method by Haarer and colleagues circumvents this by making S. cerevisiae cells in which the human beta or gamma actins replace the native ACT1 gene. This allows them to produce

© 2023. Published by The Company of Biologists under the terms of the Creative Commons Attribution License (https://creativecommons.org/licenses/by/4.0/).

human beta and gamma actin without contaminating native actin. This in my opinion is the strength. I welcome additional methods to purify actins, as a means to minimize the use of rabbit or chicken actin.

I think there are some disadvantages, which I list below. I hope the authors can address these concerns.

Comments for the author

I have two major concern.

1. The authors mention that expression of the human beta and gamma actins leads to slow growth (especially gamma). I wonder if this leads to the actin genes accumulating mutations over time? Can the authors do an experiment or discuss how they are sure that the slow growth and sickness of the strains does not lead to an evolved actin different from the original amino acid sequence.

2. Can the authors make the system more useable by perhaps expressing NAA80 and / or SETD3?

I have two minor points

1. It is important to show all mass spec data, especially the RDD data since recent work from Arnesen's lab has questioned the very existence of R actin except under very special conditions.

2. It is also important to show some images of cells carrying the beta and gamma actin genes.

First revision

<u>Author response to reviewers' comments</u>

Notes from the Editor:
As you will see, the reviewers were positive but are quite polarised in their comments, which is why we waited for the third reviewer but given the time I think it was better make a decision. As you will see reviewer 1 raises a number criticisms and suggests providing more biochemical repeats or to focus on the actual purification method and the yeast cells (a point also raised by reviewer 2). Reviewer 2 also wonders if you can improve your system by expressing NAA80 and/or SETD3. I'm sure it might help but I think that is beyond the scope of the current paper as it will require a substantial amount of additional work. I leave it up to you which route you feel you want to go and you might even want to do both but I would be happy with either. We would then return it to the reviewers.
Thank you for your very generous decision. We performed new experiments with appropriate replication and focused the text on the method. We addressed every point made by Reviewer 1 (point-by-point details are below). We are very curious about NAA80 and/or SETD3 (especially since it works very well in mammalian and *Pichia* systems!) but we did not address it in this work.

Please ensure that you clearly highlight all changes made in the revised manuscript. Please avoid using 'Tracked changes' in Word files as these are lost in PDF conversion.
There were many changes and I fear this version might be very distracting. We include this version because it was requested and a version that does not contain this mark up.

I should be grateful if you would also provide a point-by-point response detailing how you have dealt with the points raised by the reviewers in the 'Response to Reviewers' box. Please attend to all of the reviewers' comments. If you do not agree with any of their criticisms or suggestions please explain clearly why this is so.
A point-by-point response is below. We have addressed each concern, except for the NAA80/SETD3 ideas as they are beyond the scope of this work.

© 2023. Published by The Company of Biologists under the terms of the Creative Commons Attribution License (https://creativecommons.org/licenses/by/4.0/).

Reviewer 1 Comments for the Author:

While this work has the potential to be interesting and impactful, several issues are noted, specifically the lack of adequate replicates, quantifications, statistical analyses, and controls. As a result, the limited biochemical analysis of expressed actin isoforms shown harms the paper instead of enhancing its impact. There are two ways around this problem. The paper could simply focus on the expression-purification method with a more thorough analysis of the yeast strains expressing these isoforms. Alternatively, the authors could perform more replicates, quantifications, and statistical analyses. Another problem is with post-translational modification, which is poorly addressed while being a key factor for actin function. These issues are specifically developed below.
Thank you for your comments, which have substantially improved this work. The data now included in the paper has undergone more extensive analysis and now includes three or more replicates and appropriate controls. We also now more carefully address the post-translational state of the recombinant human actin purified from our system.

Major concerns:
(1) There are several issues with post-translational modification and its treatment in this paper. One of the disadvantages of yeast as an expression system for actin is that it fails to add important post-translational modifications known to impact actin's function in mammals. The authors need to state this clearly, and specifically in the following two instances: On page 4, "While actin purified by this approach may not undergo standard N-terminal processing…" should be "While actin purified by this approach does not undergo standard human N-terminal processing …". On page 12, "A potential drawback of our system is that yeast-produced actin may lack specific…" should be "… yeast-produced actin lacks specific…".
Thank you for suggesting a wording that more accurately reflects the post-translational state of these actin isoforms. We now use the wording suggested by this reviewer.

One study (PMID: 29581253) reported that the mere absence of actin's N-terminal acetyl group can lead to substantial effects on filament elongation rate for actin alone and by the formin mDia1. His73 methylation on the other hand reduces the nucleotide-exchange rate on actin and accelerates filament assembly (PMID: 30626964).
These are important points and references. We have repeated mass-spec analyses on purified proteins to improve peptide data sets. Our β- and γ-actin is acetylated on Met1, lacks methylation on His73, and more residues were arginylated compared to a skeletal muscle actin control (RMA). Further, we have now quantified the filament elongation rates of our reactions containing mDia1 and observed a significant decrease in formin-based elongation for β- and γ-actin (compared to muscle actin control). We have edited the text to highlight these important differences.

Other PTMs like phosphorylation, Lys-acetylation also affect actin's function and interactions with ABPs, but are not mentioned here. If PTMs are different for beta and gamma-1 actin (as reported here for example for N-terminal acetylation/arginylation), this raises questions about the relevance of these experiments and reported difference between these isoforms.
We did not modify the text to specifically call out to all forms of Lys-acetylation or phosphorylation. However, we now list all observed modifications in Table 1 and Table 2 and have uploaded/shared the full MS dataset to the PRIDE database (accession: PXD040174; DOI: 10.6019/PXD040174). We clarified the text to caution potential users that recombinant actins produced in our system do not perfectly recapitulate all properties of β- and γ-actin from other eukaryotic systems. We simply offer this system as an alternative to the historic use of muscle actin, with the main advantages of: a tag-less approach; a familiar model system; and reagents and tools already present in biochemistry labs.

(2) Mass spectrometry analysis reported should include representative MS2 spectra (including Y and B ions), a listing of all the observed peptides, and proper quantification of PTM peptides vs. total # of observed peptides. The proteomics data should be deposited with an appropriate database (as customary). The reported arginylation of beta actin on the 3rd residue is specifically problematic because it cannot be reliable distinguished in mass from N-terminal acetylation on the 2nd residue.
We thank this reviewer for pointing out the proper way to display and discuss mass spec (MS) data. We now include example spectra in Figure 1 and list observed modifications in Table S1 and Table

© 2023. Published by The Company of Biologists under the terms of the Creative Commons Attribution License (https://creativecommons.org/licenses/by/4.0/).

S2. We appreciated that MS datasets comparing skeletal muscle- and other forms of actin have value to the field and users of any purification system. Thus, we have uploaded and shared the full MS datasets to the PRIDE database (accession: PXD040174; DOI: 10.6019/PXD040174). We have modified our description of PTMs on the 3rd residue, better highlighting the challenge of this residue as suggested by the reviewer above.

(3) Many experiments lack replicates, quantification, statistical comparisons, and the alpha skeletal actin control. Specific comments per figures/experiments:
The first submission of this work was an attempt to summarize very old (>10 year) data that was mostly collected in a "proof-of-concept" manner. As encouraged by all reviewers we have repeated, quantified, and perform statistical comparisons to bring these observations to current publication standards (individual details below).

a.Figure 1A: The coloring for acetylation and arginylation does not match what is reported in the discussion.
We have removed the original coloring for Figure 1A. Now the only colored letters represent the residues responsible for N-terminal acetylation. This coloring is consistent with the peptide analysis in panels 1B-D.

b.Figure 1B&C: There should be some actin in the supernatant after polymerization as polymerization reaches equilibrium, but the gels are too processed to see this.
We repeated (in triplicate) the pelleting assays present in OLD Figure 1B and C. New gels, saved in a non-compressed format, are now present in NEW Figure 2A and 2B with the full gels and full gels of all replicates in NEW Figure S3. We now use α-actin (RMA) instead of yeast actin as a more appropriate control. Also, as suggested by the reviewer we have included quantification (band densitometry) of these gels in NEW Figure 2C and NEW Figure S3C.

c.Figure 1D: Pyrene-actin polymerization was conducted but not carried out to completion (plateau) and lacks proper replicates and quantification. It seems that the sole purpose of the pyrene data is to show the expressed actin polymerizes. The readers can see this in other figures (including Figure 1E&F). Additionally, it appears that gamma-1 actin may be slower than beta actin in pyrene polymerization assays, but the opposite is observed in TIRF microscopy experiments. Which experiment should we believe? Consider either removing this data or completing the experiments (at least 3 replicates that reach plateau) and quantifications of the polymerization rates.
The reviewer correctly identified the original reason for including pyrene curves in OLD Figure 1D – to simply state that each expressed actin polymerizes. Unlike other studies, this single-pass experiment was performed with 100% β- and γ-actin (including the pyrene-label). As suggested by the reviewer we have removed this figure, as we did not have enough pyrene-labeled actin (of each isoform) to perform the replicates and better methods for assessing polymerization methods are present elsewhere in the manuscript.

d.Figure 2B: Please report the values ± SEM and significance for nucleation rates in the text as done for elongation rates.
We now report the values for nucleation and elongation rates ± SE in the text. The rates associated with OLD Figure 2B-C (NEW Figure 3B-C) are listed on lines 144-149 of the revised text.

We have also calculated the elongation rates for filaments polymerized with profilin and/or the formin, mDia1. We include this information in NEW Figure 4D-F and state the rates of elongation in the revised text on lines 172-174.

e.Figure 3A: Clarify use of human cofilin-1 in methods and throughout the paper. Clarify why a yeast cofilin preparation is cited in the methods and whether it was used. Additionally, this whole experiment doesn't make much sense. Why does so much actin pellet in the G-buffer condition? A negative control showing just cofilin-1 storage buffer w/o cofilin is missing. There is little explanation of what the gels mean in the figure legend or within the text. Could it be that cofilin-1 is crashing out causing actin to crash out and pellet with it? The rabbit alpha actin control is missing, and it is not explained why yeast actin is used instead. Replicates and gel quantifications comparing the isoforms are missing. Please correct or consider removing the cofilin experiment.
We agree with this reviewer and have removed cofilin data from the manuscript.

© 2023. Published by The Company of Biologists under the terms of the Creative Commons Attribution License (https://creativecommons.org/licenses/by/4.0/).

f.Figure 3C: kD values in the text don't precisely match those in the figure.
The k_D values in the text now precisely match those in Figure 4A, with the same number of significant digits.

g.Figure 3D&E: Profilin1 experiments should be quantified with at least three replicates, and the alpha skeletal actin control added.
We performed (in triplicate) new assays with each actin (α-, β-, and γ-) in the presence of profilin to replace the panels of OLD Figure 3D-E. New gels, saved in a non-compressed format, are now present in NEW Figure 4B with the full gels and full gels of all replicates in NEW Figure S4A-C. We use α -actin (RMA) instead of yeast actin as a more appropriate control and have performed profilin alone controls in F- or G-buffer. Also, as suggested by the reviewer we have included quantification (band densitometry) of these gels in NEW Figure 4C.

h.Figure 3F&G and Movie: Formin mDia1 nucleates and elongates actin filaments. Therefore, TIRF quantification should be conducted for nucleation and elongation and ± profilin-1. At least three replicates and statistical comparisons should be performed for each experiment, including controls with alpha skeletal actin.
To address this concern, we performed TIRF assays (in triplicate) with each actin alone (α-, β-, and γ-actin), each in the presence of 2 μM profilin-1, each in the presence of 10 nM mDia1(FH1-C), and each with profilin-1 and mDia1(FH1-C), combined (in NEW Figure 4D-F and NEW Movie 2). We measured the number of filaments present in fields of view as a readout of actin filament nucleation (no statistically significant differences were found). We also measured the elongation rate of individual filaments present in these reactions (5-15 filaments measured per movie, 15-45 total filaments across replicates of each condition). We observed a significant reduction in the elongation rate of β- and γ-actin filaments compared to α-actin (RMA) controls ($P \leq 0.0012$), but not different compared to each other ($P > 0.99$)(NEW Figure 4D).

i.Figure 4B&C: The figure legend does not indicate the number of independent experiments, and it looks like in some cases n=2, which is insufficient for statistical comparison.

Additional experiments and analysis were performed to increase the sample size to n >=3 (shown as dots) for the assays shown in NEW Figure 5. As requested we now include information on n, the statistical test used, and statistical comparisons in the legends of each figure.

Minor comments:
(1) Yeast expressing alpha-actin as the sole actin did not survive. Please, cite PMID: 16118223, which made a similar observation.
Thank you for this information! We now cite the suggested reference in addition to PMID: 16118223, where authors first cite this finding as an unpublished observation.

(2) Most gels are overly processed and look fake.
As stated above, the original images were constructed using gels that were not saved in an uncompressed format. We have re-run each gel (and replicate), replaced scans with high resolution uncompressed images, and provide full images of gels used for analyses in Figures S3 and S4.

(3) Report significant differences with brackets and asterisks (or P values) above the graphs, according to common practice.
We now include brackets to help orient the reader to the different statistical comparisons performed throughout the manuscript. We also state details of the statistical analyses performed in the methods. We use the standard $P \leq 0.05$ threshold to denote significance throughout.

(4) Include gels in supplementary information of all the ABPs used.
In NEW Figure S5 we include a gel showing the purity of all actin isoforms and actin regulatory proteins used in TIRF assays.

(5) Seems statistics were conducted using one-way ANOVA with Tukey post-hoc analysis, but this is not always indicated.

© 2023. Published by The Company of Biologists under the terms of the Creative Commons Attribution License (https://creativecommons.org/licenses/by/4.0/).

The reviewer is correct. We use one-way ANOVA with Tukey post-hoc analysis throughout the manuscript. We now explicitly state ANOVA as the test being performed in each figure legend and explicitly state this in the NEW “Data analysis, statistics, and availability” section in the methods.

(6) A broad range of critical concentrations is reported (0.15 to 0.25μM), but the approach to calculate critical concentrations is not described. Either remove this information or precisely show how Cc was calculated.
The critical concentrations mentioned previously were calculated by end-point fluorescence measurements in pyrene assays. As stated earlier these assays were not appropriate as the measurements did not reach the plateau and due to insufficient replication. We removed this information from the text.

(7) The authors report “high yields”, but 0.5-1 mg per L yeast culture, while adequate, cannot be considered high yield. Just listing the numbers is sufficient.
Thank you. We now state the value without any additional qualifying remarks.

Reviewer 2 Advance Summary and Potential Significance to Field:
The manuscript by Haarer and colleagues reports a new approach to purifying actin isoforms. Actin isoforms have been difficult to purify traditionally, although over the last decade the groups of Uyeda, Trybus, Lappalainen, and Balasubramanian have developed a number of systems (in Dicty, Pichia, sf9 cells), largely based on thymosinb4 binding and subsequent purification and chymotrypsin cleavage. The one slight complication is the potential to copurify small amounts of native actin.

The current method by Haarer and colleagues circumvents this by making S. cerevisiae cells in which the human beta or gamma actins replace the native ACT1 gene. This allows them to produce human beta and gamma actin without contaminating native actin. This in my opinion is the strength. I welcome additional methods to purify actins, as a means to minimize the use of rabbit or chicken actin.
Thank you for recognizing this strength! We agree that our tag-less approach to obtaining actin isoforms lacking contamination with “host” actin may be useful in some contexts.

I think there are some disadvantages, which I list below. I hope the authors can address these concerns.

Reviewer 2 Comments for the Author:
I have two major concern.
1. The authors mention that expression of the human beta and gamma actins leads to slow growth (especially gamma). I wonder if this leads to the actin genes accumulating mutations over time? Can the authors do an experiment or discuss how they are sure that the slow growth and sickness of the strains does not lead to an evolved actin different from the original amino acid sequence.
To address concerns of potential mutation of plasmid-borne actin sequences in host yeast strains, we re-isolated and sequenced plasmids from the β- and γ-actin strains at the time of purification. In each case, actin gene sequences were identical to the starting yeast-optimized *ACTB* and *ACTG1*. In the event other readers have this concern, we now include this information in the methods section (lines 265-267).

2. Can the authors make the system more useable by perhaps expressing NAA80 and / or SETD3?
Mass spec analyses revealed that the actin produced in our system is often acetylated on Met-1 but His73 is not methylated. This reviewer’s idea is very interesting especially given the success of expressing NAA80 and SETD3 in other expression systems. However, these experiments are beyond the current scope of this work.

I have two minor points
1. It is important to show all mass spec data, especially the RDD data since recent work from Arnesen's lab has questioned the very existence of R actin, except under very special conditions.
We now include example MS spectra (Figure 1B-D), tables of modified residues (Table S1 and Table S2), and we have deposited the full dataset in the PRIDE database (accession: PXD040174; DOI: 10.6019/PXD040174). The helpful reference this reviewer provided clued us into some of the

© 2023. Published by The Company of Biologists under the terms of the Creative Commons Attribution License (https://creativecommons.org/licenses/by/4.0/).

difficulties associated with reliably determining arginylation. Thus, we removed emphasis on N-terminal arginylation and listed the software generated peak identities. Intriguingly, both β- and γ-actin have arginylated peaks not found in the α-actin (RMA) control. We are more transparent about this finding in the discussion (lines 219-228).

2. It is also important to show some images of cells carrying the beta and gamma actin genes.
We now include representative DIC, phalloidin, and DAPI images of yeast strains (WT yeast and yeast expressing human β- or γ-actin) in New Figure S2.

Second decision letter

MS ID#: JOCES/2022/260540

MS TITLE: Purification of Human β- and γ-actin from Budding Yeast

AUTHORS: Brian K Haarer, Morgan L Pimm, Ebbing P deJong, Jessica L Henty-Ridilla, and David C Amberg
ARTICLE TYPE: Tools and Resources

Its taken longer than it should as we have been chasing reviewer 2 for some time but we have now reached a decision on the above manuscript.

To see the reviewers' reports and a copy of this decision letter, please go to: https://submit-jcs.biologists.org and click on the 'Manuscripts with Decisions' queue in the Author Area. (Corresponding author only has access to reviews.)

As you will see, the reviewers gave favourable reports but reviewer 1 raises some minor points that will require amendments to your manuscript. Its easier to do it now than at the proof stage. I hope that you will be able to carry these out because I would like to be able to accept your paper.

Please ensure that you clearly highlight all changes made in the revised manuscript. Please avoid using 'Tracked changes' in Word files as these are lost in PDF conversion.

I should be grateful if you would also provide a point-by-point response detailing how you have dealt with the points raised by the reviewers in the 'Response to Reviewers' box. Please attend to all of the reviewers' comments. If you do not agree with any of their criticisms or suggestions please explain clearly why this is so.

Reviewer 1

Advance summary and potential significance to field

This paper by Haarer et al. has improved substantially during revisions. In principle, I support publication of this work in Journal of Cell Science, but there are some issues that remain to be addressed.

Comments for the author

1. Differences among actin isoforms in several figures of the paper are deemed significant, but P values are not provided. P values were apparently calculated but are not shown. It would be very helpful to display P values right on the figures, and there is no reason why not to do it (preferably actual numbers and not asterisks). This concerns Figs. 3C, 4E, 4F, 5B, 5C. Additionally, the Y axis in Fig. 4F must be expanded so that the differences in “Actin alone” become clear.

2. There is still significant inconstancy in the way N-arginylation is treated in this paper. N-arginylation is mentioned in the Introduction, then brought about in the Results as a possibility, which is immediately discarded due to lack of reliable data. But then it is mentioned again in the

© 2023. Published by The Company of Biologists under the terms of the Creative Commons Attribution License (https://creativecommons.org/licenses/by/4.0/).

Discussion in a weird and inconsistent manner “we observed precocious arginylation of beta and gamma actin purified from yeast sources compared to actin purified from muscle (Tables S1 and S2)". What is “precocious arginylation”? Moreover, this is not consistent with the Results where it is concluded “given the difficulties of reliably determining protein arginylation (Xu et al., 2009), we are uncertain if this modification …” Why insist on this point, which only serves to weaken the paper? Simply put, N-arginylation of beta actin on the 3rd residue cannot be reliably set apart by mass spectrometry from N-acetylation on the 2nd residue (which is what everybody believes and observes, even in structures). Why is that other actin isoforms are not N-arginylated? Because the masses would be sufficiently different, and this confusing situation would not be possible. N-arginylation has not been reproducibly identified but by one lab.

Reviewer 2

Advance summary and potential significance to field

I am happy with the revisions.

Comments for the author

I am happy with the revisions made.

Second revision

Author response to reviewers' comments

*A nicely formatted version is uploaded with the Supplementary Information. What follows is the requested copy/paste response:

Notes from the Editor:
As you will see, the reviewers gave favourable reports but reviewer 1 raises some minor points that will require amendments to your manuscript. Its easier to do it now than at the proof stage. I hope that you will be able to carry these out because I would like to be able to accept your paper.
Thank you! We would also very much like our paper to be accepted. We have addressed all the reviewer comments below.

Please ensure that you clearly highlight all changes made in the revised manuscript. Please avoid using 'Tracked changes' in Word files as these are lost in PDF conversion.
We have uploaded a version with changes highlighted and an unmarked version for ease.

I should be grateful if you would also provide a point-by-point response detailing how you have dealt with the points raised by the reviewers in the 'Response to Reviewers' box. Please attend to all of the reviewers' comments. If you do not agree with any of their criticisms or suggestions please explain clearly why this is so.
We believe we have attended to all the reviewer’s comments in the point-by-point dialog, below.

Reviewer 1:
This paper by Haarer et al. has improved substantially during revisions. In principle, I support publication of this work in Journal of Cell Science, but there are some issues that remain to be addressed.
Thank you! You gave us a lot of very thoughtful feedback in the first round, and we have taken your advice on the issues that remained, below.

1. Differences among actin isoforms in several figures of the paper are deemed significant, but P values are not provided. P values were apparently calculated but are not shown. It would be very helpful to display P values right on the figures, and there is no reason why not to do it (preferably actual numbers and not asterisks). This concerns Figs. 3C, 4E, 4F, 5B, 5C. Additionally, the Y axis in Fig. 4F must be expanded so that the differences in “Actin alone” become clear.

© 2023. Published by The Company of Biologists under the terms of the Creative Commons Attribution License (https://creativecommons.org/licenses/by/4.0/).

In the previous version, we placed all calculated p-values in the text (that were significant) and stated in the methods the cut-offs we used (the standard: $p<0.05$ = significant; if $p>0.05$, not). We strongly feel that adding the p-values above all comparisons ruins the aesthetic of our figures and is not consistent with the requirements of the journal, but we did this to appease this reviewer. We have one exception, Figure 4F, where including the values made the figure too crowded. Thus, to satisfy this reviewer we now offer **new Supplemental Figure 5** that plots the exact data present in 4F next to a rescaled version appropriate for the actin alone controls. We also include a table with relevant statistical comparisons (the p-value shown in the figures is the comparison for ALL conditions). This new supplemental figure also solves our uncertainty on how to change the Y- axis of this panel (also requested) without misrepresenting the data. Notably, the controls in question are *additional* replicates of the data presented in Figure 3C, where the differences can be clearly visualized.

2. There is still significant inconstancy in the way N-arginylation is treated in this paper. N-arginylation is mentioned in the Introduction, then brought about in the Results as a possibility, which is immediately discarded due to lack of reliable data. But then it is mentioned again in the Discussion in a weird and inconsistent manner “we observed precocious arginylation of beta and gamma actin purified from yeast sources compared to actin purified from muscle (Tables S1 and S2)". What is “precocious arginylation”? Moreover, this is not consistent with the Results where it is concluded “given the difficulties of reliably determining protein arginylation (Xu et al., 2009), we are uncertain if this modification …” Why insist on this point, which only serves to weaken the paper? Simply put, N-arginylation of beta actin on the 3rd residue cannot be reliably set apart by mass spectrometry from N-acetylation on the 2nd residue (which is what everybody believes and observes, even in structures). Why is that other actin isoforms are not N-arginylated? Because the masses would be sufficiently different, and this confusing situation would not be possible. N-arginylation has not been reproducibly identified but by one lab.
We appreciate these comments about N-arginylation and clearly we have struggled with how to talk about it in our work. By no means are we interested in taking any stance on the arginylation situation mentioned by this reviewer. We have adjusted the language in the results and discussion for consistency (the intro merely mentions that this system could be modified to produce such a version of actin so we left it). We also use your suggested language to handle N-arginylation on ß-actin – thank you!

Reviewer 2
I am happy with the revisions.
Thank you, we appreciate your thoughtful suggestions from the first-round and support.

Third decision letter

MS ID#: JOCES/2022/260540

MS TITLE: Purification of Human ß- and γ-actin from Budding Yeast

AUTHORS: Brian K Haarer, Morgan L Pimm, Ebbing P deJong, David C Amberg, and Jessica L Henty-Ridilla
ARTICLE TYPE: Tools and Resources

I am happy to tell you that your manuscript has been accepted for publication in Journal of Cell Science, pending standard ethics checks.

© 2023. Published by The Company of Biologists under the terms of the Creative Commons Attribution License (https://creativecommons.org/licenses/by/4.0/).
